# Rational design of crystalline supermicroporous covalent organic frameworks with triangular topologies

**DOI:** 10.1038/ncomms8786

**Published:** 2015-07-16

**Authors:** Sasanka Dalapati, Matthew Addicoat, Shangbin Jin, Tsuneaki Sakurai, Jia Gao, Hong Xu, Stephan Irle, Shu Seki, Donglin Jiang

**Affiliations:** 1 Department of Materials Molecular Science, Institute for Molecular Science, National Institutes of Natural Sciences, 5-1 Higashiyama, Myodaiji, Okazaki 444-8787, Japan.; 2 Department of Chemistry, WPI-Research Initiative Institute of Transformative Bio-Molecules, Graduate School of Science, Nagoya University, Furo-cho, Chikusa-ku, Nagoya 464-8602, Japan.; 3 Department of Molecular Engineering, Graduate School of Engineering, Kyoto University, A4 Kyoto University Katsura Campus, Nishikyo-ku, Kyoto 615-8510, Japan.

## Abstract

Covalent organic frameworks (COFs) are an emerging class of highly ordered porous polymers with many potential applications. They are currently designed and synthesized through hexagonal and tetragonal topologies, limiting the access to and exploration of new structures and properties. Here, we report that a triangular topology can be developed for the rational design and synthesis of a new class of COFs. The triangular topology features small pore sizes down to 12 Å, which is among the smallest pores for COFs reported to date, and high π-column densities of up to 0.25 nm^−2^, which exceeds those of supramolecular columnar π-arrays and other COF materials. These crystalline COFs facilitate π-cloud delocalization and are highly conductive, with a hole mobility that is among the highest reported for COFs and polygraphitic ensembles.

## Abstract (web summary)

Covalent organic frameworks are currently arousing considerable interest due to their desirable properties for a wide range of applications. Here, Jiang *et al*. report two such materials with triangular topologies which exhibit high hole mobility arising from extensive π-cloud delocalisation.

## Graphical abstract

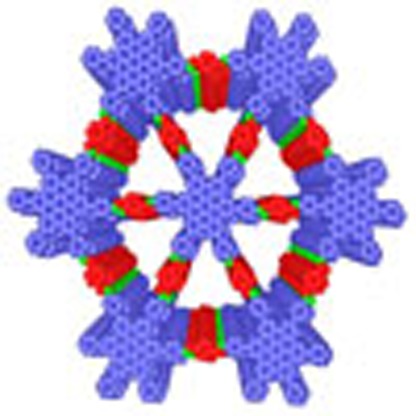

Covalent organic frameworks (COFs) are crystalline porous polymers with periodic ordering of organic building blocks^1^,^2^,^3^,^4^ and exhibit great potential for applications in gas storage^5^,^6^, manufacture of semiconductive and photoconductive devices^7^,^8^,^9^,^10^,^11^,^12^, energy conversion^10^,^12^,^13^ and storage^14^,^15^, and heterogeneous catalysis^16^,^17^,^18^,^19^. The key to the design of COFs is their topology diagram, which defines the polygon size and shape and determines the crystal lattice. Hexagonal and tetragonal topologies have been exploited for the design of two-dimensional (2D) COFs with discrete pores and ordered skeletons^1^,^2^,^3^,^4^,^5^,^6^,^7^,^8^,^9^,^10^,^11^,^12^,^13^,^14^,^15^,^16^,^17^,^18^,^19^,^20^,^21^,^22^,^23^,^24^,^25^,^26^,^27^,^28^,^29^,^30^,^31^,^32^,^33^,^34^. The introduction of new topologies is highly desired to broaden the structural diversity and application of COF materials.

The triangular topology consists of *C*_6_-geometric vertices, which is the highest symmetry of a benzene system and has a potential to efficiently exploit space for titling organic units into a crystal lattice. With this geometry, a triangular COF would feature the smallest pore size and most dense π-columns among all of the 2D COF materials. Heine *et al*. theoretically predicted the presence of a triangular COF structure in 2010 (ref. 30). However, the triangular topology has not been reported for the practical synthesis of COFs.

Here, we report on the rational design and synthesis of a new class of COFs based on the triangular topology. We demonstrate the synthesis of two triangular COFs (defined as HPB-COF and HBC-COF) by using two different *C*_6_-symmetric vertices—one is hexaphenylbenzene (HPB), which is a typical propeller-shaped π-unit, and the other is hexabenzocoronene (HBC), which is a large graphitic π-unit—for Schiff-base polymerization with *C*_2_-symmetric benzene linkers. These triangular COFs form supermicropores with pore sizes of as low as 12 Å, which is among the lowest reported for COFs, whereas the density of π-columns can reach 0.25 nm^−2^, which exceeds those of the COFs and supramolecular π-arrays reported to date. These crystalline COFs exhibit excellent thermal and solvent stabilities. We demonstrate that the triangular COFs enable both intra- and inter-layer π-cloud delocalization and exhibit a prominent photoconductivity, with carrier mobilities as high as 0.7 cm^2^ V^−1^ s^−1^, which is among the highest reported for 2D COFs and polygraphitic ensembles. These results suggest that the triangular topology is useful for designing COFs with unique structures to be used in a wide variety of applications, such as gas storage, catalysis and the manufacture of sensing and semiconducting devices.

## Results

### Design and features of the triangular topology

**Figure 1:**
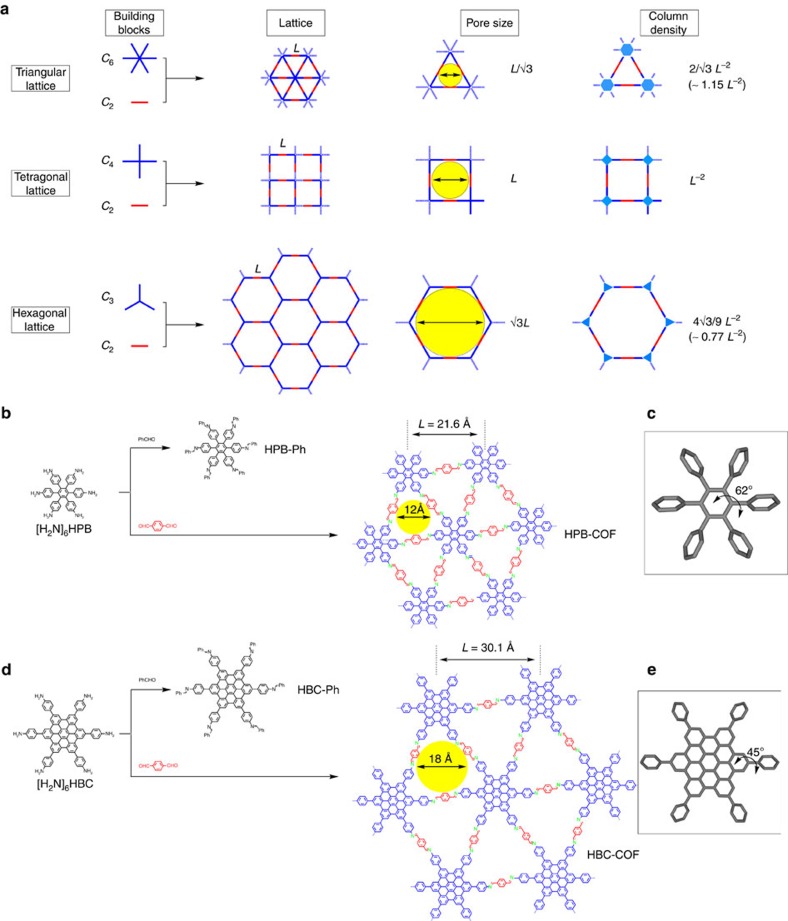
Design of topology diagrams and synthesis of trigonal COFs. (**a**) Topology diagrams for COFs and their pore size and π-column density. (**b**) Schematic representation of the synthesis of imine-linked triangular HPB-COF, together with its model reaction. (**c**) Propeller-shaped HPB building block. (**d**) Schematic representation of the synthesis of imine-linked triangular HBC-COF, together with its model reaction. (**e**) HBC building block.

Figure 1 shows the topology diagrams used for designing the 2D COFs. The triangular topology features the smallest pore size and highest π-column density among all of the topologies. The hexagonal topology leads to a pore size of √3*L* (∼1.732*L*), whereas the tetragonal topology yields a pore size of *L*, as the length between two vertices is defined as *L* (Fig. 1a). By contrast, the triangular topology generates a smaller pore diameter of only *L*/√3 (∼0.58*L*), which is approximately half and one-third of those of the tetragonal and hexagonal COFs, respectively. This is the reason why the 2D COFs synthesized to date using the hexagonal and tetragonal topologies typically possess large pores with sizes in the mesoporous range (>2 nm). The introduction of a topology that directly forms microporous COFs (pore size<2 nm) is highly desired. The microporous COFs are useful for gas storage^35^. We demonstrate that the triangular topology offers a straightforward strategy for the synthesis of microporous COFs. Another character of the triangular topology is that it provides the highest density of the π-columns among 2D COFs (Fig. 1a). The density of the π-columns is 2/√3*L*^−2^ (∼1.15*L*^−2^), *L*^−2^ and 4√3/9*L*^−2^ (∼0.77*L*^−2^), for the triangular, tetragonal and hexagonal topologies, respectively. Semiconducting and optoelectronic devices prefer ordered and dense π-units to achieve high performance.

Two *C*_6_-symmetric molecules, that is, [H_2_N]_6_HPB and [H_2_N]_6_HBC, bearing six amino groups were synthesized as the building blocks for the vertices of triangular COFs. [H_2_N]_6_HPB is a typical propeller-shaped molecule with a twisted angle of 62° between the six phenyl units and focal benzene ring (Fig. 1c). [H_2_N]_6_HBC is a graphitic molecule with a less twisted angle of 45° between the six phenyl groups and focal HBC moiety (Fig. 1e). [H_2_N]_6_HPB, [H_2_N]_6_HBC and their intermediates were unambiguously characterized by NMR spectroscopy and mass spectrometry (Supplementary Figs 1–9). The amino groups of [H_2_N]_6_HPB and [H_2_N]_6_HBC reacted with benzaldehyde to form imine linkages (Fig. 1b,d, Supplementary Methods). By replacing the monofunctional benzaldehyde with a bifunctional aldehyde, that is, *C*_2_-symmetric terephthalaldehyde, polymerization with [H_2_N]_6_HPB or [H_2_N]_6_HBC yielded a triangular COF, in which the HPB or HBC unit occupies the vertices of imine-linked triangular polygons (Fig. 1b,d). HPB-COF and HBC-COF are supermicroporous polymers with pore sizes of 1.2 and 1.8 nm, respectively (Fig. 1b,d). The triangular topology yields a π-column density of as high as 0.25 nm^−2^ (Table 1), which is the highest among 2D COFs and also exceeds that of supramolecular columnar π-arrays^35^,^36^,^37^.

### Synthesis and characterization

HPB-COF and HBC-COF (Fig. 1b,d) were synthesized via the polymerization of terephthalaldehyde with [H_2_N]_6_HPB and [H_2_N]_6_HBC under solvothermal conditions. We screened reaction conditions, including solvent, catalyst concentration, reaction temperature and reaction time (Supplementary Figs 10–12, Supplementary Tables 1 and 2). For the optimal conditions, HPB-COF was synthesized in toluene/3 M AcOH (20/1 v/v) at 120 °C for 9 days and obtained as yellow solid in a 70% isolated yield. HBC-COF was synthesized in dioxane/*n*-butanol/6 M AcOH (19/1/1 v/v) at 120 °C for 12 days and obtained as deep red powder in a 95% isolated yield. HPB-COF and HBC-COF were unambiguously characterized by various analytical methods (Supplementary Figs 10–21).

Infrared spectroscopy exhibited a typical stretching vibration band at 1,622–1,626 cm^−1^, which was assigned to the C=N bond and also confirmed by the model compounds of HPB-Ph and HBC-Ph (Supplementary Figs 13 and 14). HPB-COF exhibited a micrometre-scale sheet-shape morphology, as revealed by field-emission scanning electron microscopy, whereas HBC-COF adopted a particle morphology (Supplementary Fig. 15). High-resolution transmission electron microscopy revealed the presence of layered and triangular textures (Supplementary Fig. 16).

### Crystal structure

**Figure 2:**
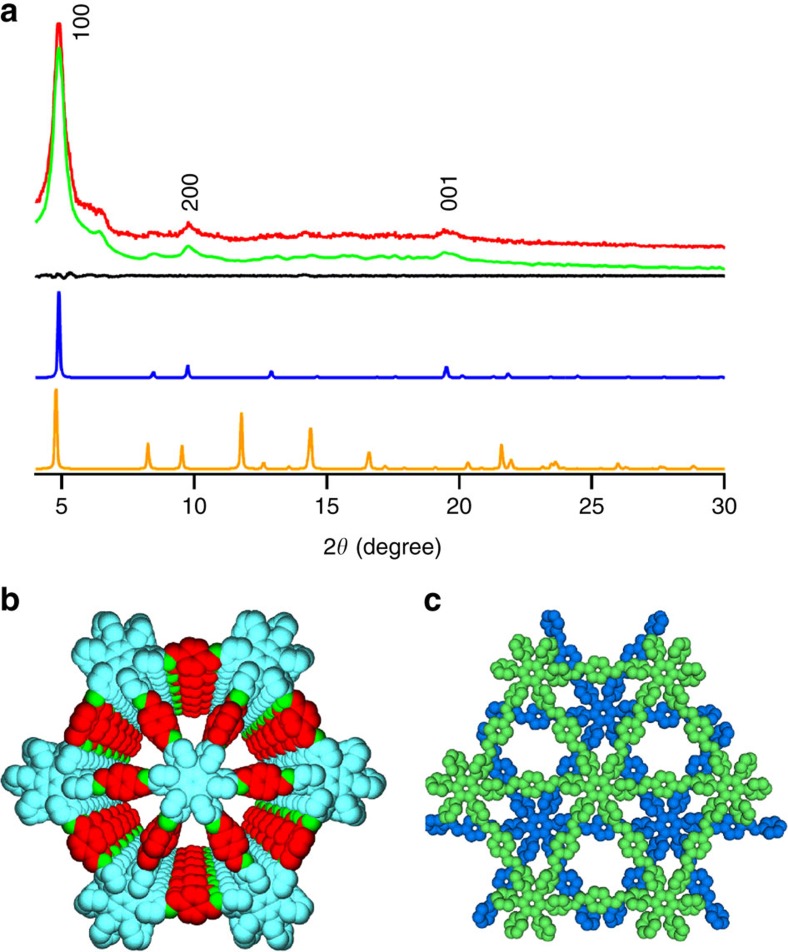
Crystal structure of HPB-COF. (**a**) XRD patterns of experimentally observed (red curve), Pawley refined pattern (green curve), their difference (black curve), eclipsed hybrid AA stacking mode (blue curve) and staggered AB stacking mode (orange curve). The crystal facets are shown with indices on the peaks. (**b**) View of the eclipsed hybrid AA stacking structure. (**c**) View of the staggered AB stacking structure.

**Figure 3:**
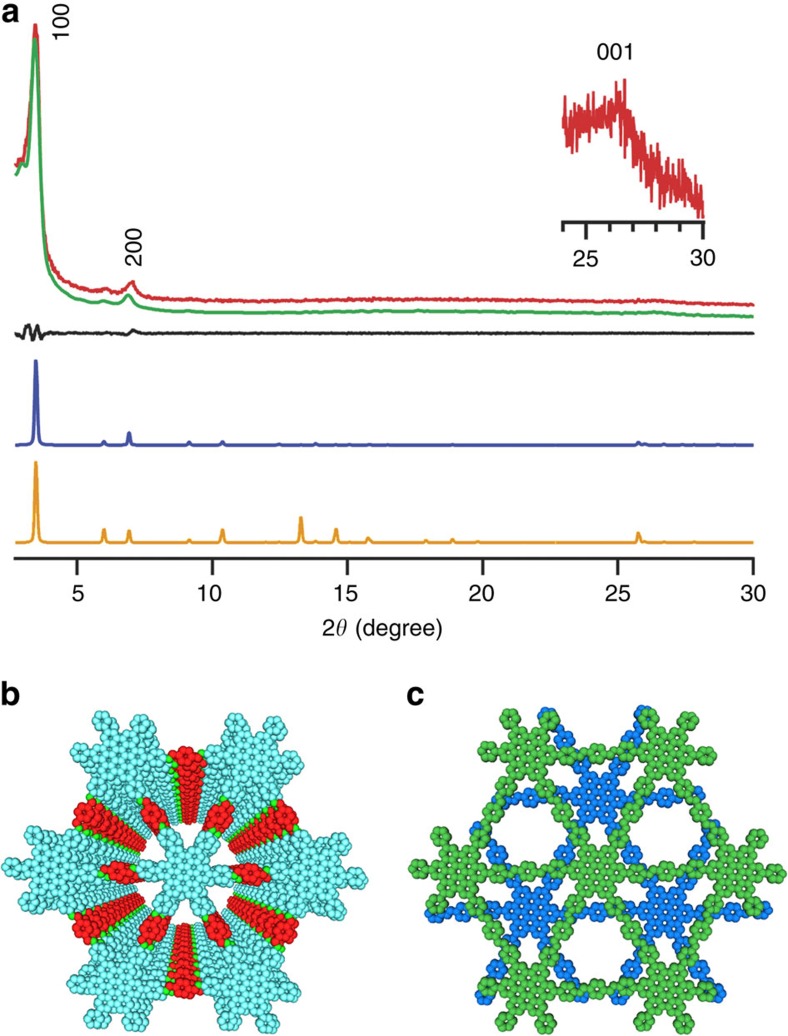
Crystal structure of HBC-COF. (**a**) XRD patterns of experimentally observed (red curve), Pawley refined pattern (green curve), their difference (black curve), 0.8 Å slipped AA stacking mode (blue curve) and staggered AB stacking mode (orange curve). The crystal facets are shown with indices on the peaks. (**b**) View of the 0.8 Å slipped AA stacking structure. (**c**) View of the staggered AB stacking structure.

Crystal structures of HPB-COF and HBC-COF were resolved by using X-ray diffraction (XRD) measurements in conjunction with Pawley refinements and structural simulations (Figs 2 and 3 and Supplementary Methods)^38^,^39^,^40^. HPB-COF exhibited XRD signals at 4.85° and 9.70°, which were assigned to the (100) and (200) facets, respectively (Fig. 2a, red curve). The presence of the (001) facet at 19.45° indicates that the periodicity of the 2D sheets is extended to the third dimension. The Pawley refinements (green curve) reproduced the experimentally observed XRD pattern with a negligible difference (black curve), confirming the correctness of the above peak assignments. HBC-COF exhibited XRD peaks at 3.4°, 6.8° and 26.26° that were assigned to the (100), (200) and (001) facets, respectively (Fig. 3a, red curve). The Pawley refinements yielded an XRD pattern (green curve) that is consistent with the observed XRD profile. The negligible difference (black curve) assures the suitable assignment of the observed XRD signals.

To generate the optimal crystal structures, the density-functional tight-binding (DFTB) method including Lennard–Jones dispersion was applied to both COFs (for details, see Supplementary Methods; http://www.dftb.org)^40^. For the monolayer of HPB-COF, the optimal cell length was *a*=*b*=21.57 Å, while the six phenyl units of HPB are twisted by 62° with respect to the central benzene ring in a propeller-like arrangement. By contrast, the monolayer of HBC-COF exhibited an optimal cell length of *a*=*b*=30.14 Å, while the six phenyl units are twisted to form a 45° angle with respect to the HBC plane. Using these monolayer structures, three stacked configurations, that is, eclipsed AA (hybrid), slipped AA and staggered AB modes, were generated and optimized. The interlayer stacking distances for the central ring, and the corresponding Lennard–Jones dispersion, and crystal stacking energies per monolayer of each structure are shown in the Supplementary Tables 3 and 4. For HPB-COF, the eclipsed hybrid AA stacking mode, which is situated as a tilted and planar arrangement with respect to the linkers for alternate layers, gives rise to the most stable structure as a result of favourable C–H···π interactions between two layers. For HBC-COF, the 0.8 Å slipped AA stacking mode is the most stable structure. The twisted angles of the phenyl groups in the HPB and HBC units decreased to 54° and 20° for the eclipsed hybrid AA mode of HPB-COF and slipped AA mode of HBC-COF, respectively. Therefore, in the frameworks, the 2D polygon sheets become more planar as a result of interlayer π–π forces. According to these structures, HPB-COF consists of HPB π-columns with a density of as high as 0.25 nm^−2^, and HBC-COF exhibits a graphitic column density of 0.13 nm^−2^ (Table 1). These densities are the highest among COFs and exceed that of supramolecular columnar HPB and HBC π-arrays. Such a highly dense yet ordered π-structure is intriguing for optoelectronic applications.

On the basis of the above optimal stacking structures, we deduced the crystal structures using the Reflex module package in Materials Studio^39^. The generated XRD patterns of the eclipsed hybrid AA stacking mode for HPB-COF (Fig. 2a, blue curve) and the 0.8 Å-slipped AA stacking mode for HBC-COF (Fig. 3a, blue curve) can accurately reproduce both the positions and intensities of the experimentally observed XRD peaks. Figures 2b and 3b illustrate the eclipsed hybrid AA and 0.8 Å-slipped AA stacking structures of HPB-COF and HBC-COF, respectively. In contrast to the AA modes, the staggered AB modes (Figs 2a and 3a, orange curves) cannot reproduce the experimentally observed XRD patterns and half of the pores are covered by the neighbouring sheets (Figs 2c and 3c).

The *C*_6_-symmetric vertices play a vital role in controlling the interlayer interactions. For example, the HPB-COF with propeller-shaped HPB vertices has an interlayer distance of 5.17 Å, which is considerably larger than that (3.54 Å) of HBC-COF. Although HPB-COF has *a* and *b* lengths that are smaller than HBC-COF, the total crystal stacking energy of HPB is calculated to be only 46.74 kcal mol^−1^ per monolayer (Table 1), which is only one-third that of HBC-COF (136.37 kcal mol^−1^). Thus, the propeller-shaped HPB knots offer a loose stacking, whereas the graphitic units enable a tight layering of 2D sheets.

### Gas adsorption and porosity

**Figure 4:**
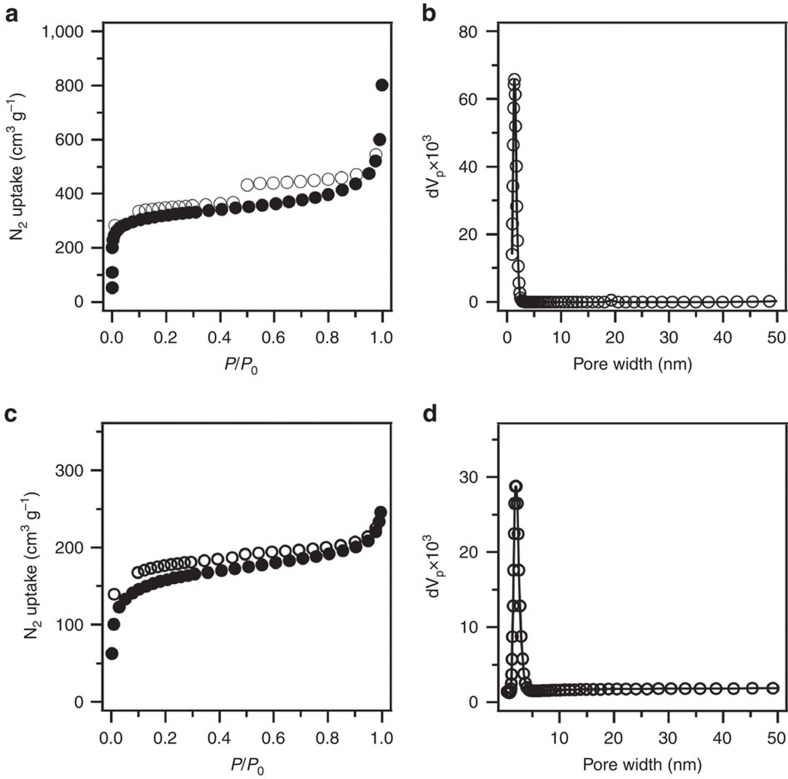
Gas adsorption. (**a**) Nitrogen sorption isotherm curve of HPB-COF (filled circles for adsorption and open circles for desorption). (**b**) Pore size distribution profile of HPB-COF. (**c**) Nitrogen sorption isotherm curve of HBC-COF (filled circles for adsorption and open circles for desorption). (**d**) Pore size distribution profile of HBC-COF.

To investigate the porous structure, nitrogen sorption isotherms were measured at 77 K (Fig. 4). HPB-COF and HBC-COF exhibited typical type I isotherms (Fig. 4a,c), which are characteristic of microporous materials. The Brunauer–Emmett–Teller (BET) surface area and pore volume for HPB-COF were calculated to be 965 m^2^ g^−1^ and 0.79 cm^3^ g^−1^, respectively. HBC-COF exhibited a BET surface area and pore volume of 469 m^2^ g^−1^ and 0.29 cm^3^ g^−1^, respectively. On the basis of the nonlocal density-functional theory calculation from the sorption curves, the pore size of HPB-CPF and HBC-COF was estimated to be 1.2 and 1.8 nm, respectively (Fig. 4b,d). These supermicropores are among the smallest reported for 2D COFs.

We calculated the theoretical BET surface areas of HPB-COF and HBC-COF using the model structures of DFTB calculations and observed that HPB-COF (Supplementary Fig. 17) and HBC-COF (Supplementary Fig. 18) have theoretical surface areas of 1,588 and 1,381 m^2^ g^−1^, respectively. The difference between experimentally observed BET surface areas and theroretical values is related to the crystallinity of the COF samples. The high theoretical surface area of HPB-COF compared with that of HBC-COF is cuased by a large *c* value (5.1 Å for HPB-COF and 3.5 Å for HBC-COF) and a low monomer molecular weight (624 for the HPB monomer versus 1,068 for the HBC monomer)^41^. These factors also reflect in the experimentally obsvered porosity.

### Stability

**Figure 5:**
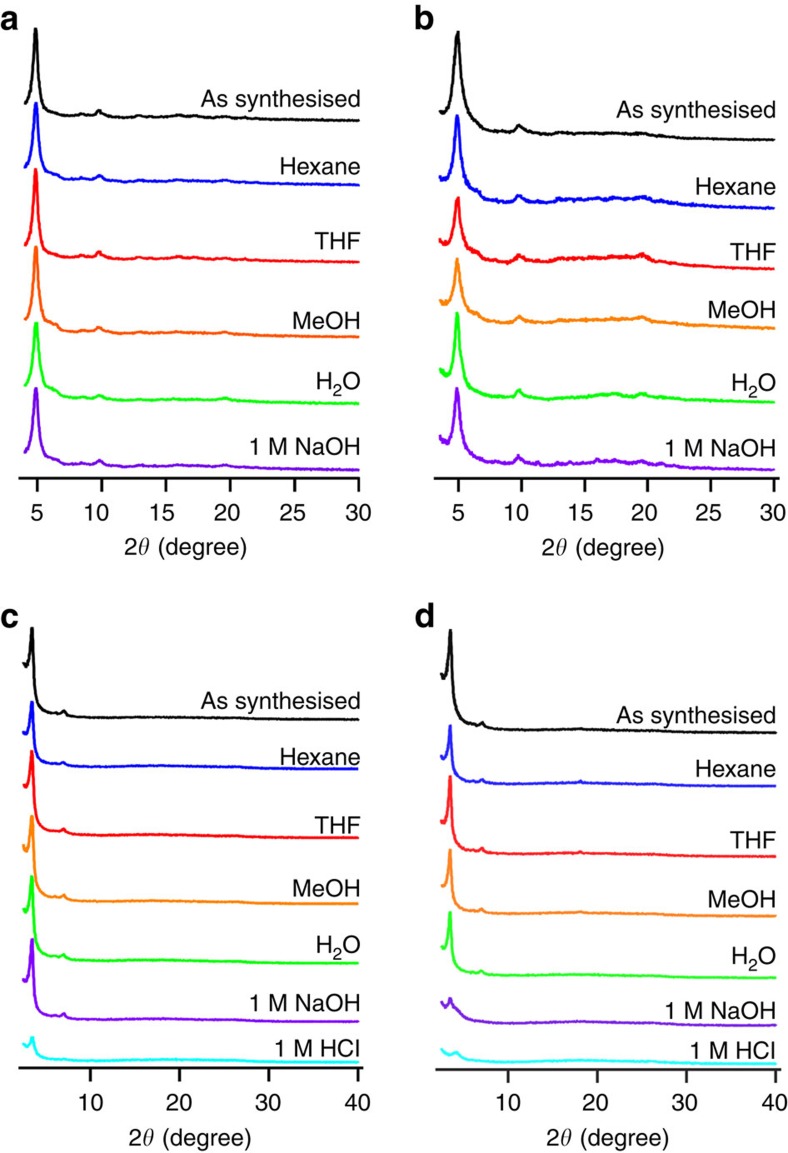
Stability. (**a**,**b**) XRD patterns of HPB-COF upon 1-day treatment under different conditions at (**a**) 25 °C and (**b**) boiling temperatures (heating at 100 °C). (**c**,**d**) XRD patterns of HBC-COF upon 1-day treatment under different conditions at (**c**) 25 °C and (**d**) boiling temperatures (heating at 100 °C).

To examine the chemical stability, we dispersed the COF samples in different solvents such as hexane, tetrahydrofuran (THF), MeOH, water and aqueous HCl (1 M) and NaOH (1 M) solutions at 25 °C and boiling temperatures (heating at 100 °C) for 24 h. Both COFs fairly retained their crystallinity (Fig. 5) and porosity (Supplementary Tables 5 and 6 and Supplementary Figs 19 and 20) upon treatment in organic solvents, such as hexane, THF and MeOH, and water at 25 °C and their boiling temperatures. Nevertheless, HBC-COF was unstable in aqueous HCl solution (1 M) at 25 and 100 °C, as revealed by their XRD and nitrogen sorption measurements. HPB-COF was much easier to be destroyed in aqueous HCl solution (1 M); no solid sample remained after treatment. Notably, HPB-COF and HBC-COF were stable in aqueous NaOH (1 M) solution at 25 °C (Fig. 5). HPB-COF kept crystallinity and porosity upon treatment in the NaOH solution at 100 °C, whereas HBC-COF was unstable in the NaOH solution at elevated temperature. The instability is likely related to the hydrolysis of the imine linkages. We conducted thermal gravimetric analysis to investigate thermal stability and did not observed gaseous decomposition products for HPB-COF and HBC-COF up to 520 and 550 °C, respectively (Supplementary Fig. 21). The stability of COFs is crucial for use in various applications.

## Discussion

We reported the first example of a semiconducting TP-COF in 2008 (ref. 7). In recent years, we and other groups have prepared a series of conducting COFs with ordered π-columns^8^,^9^,^10^,^11^,^12^,^21^,^22^,^23^,^24^,^25^,^26^,^31^,^32^. Compared with self-assembled systems and liquid crystals, a distinct feature of COFs' π-columns is that they trigger significant electronic coupling allowing π-cloud delocalization, which facilitates charge transport through the π-channels. However, except for a few examples^10^,^26^, the majority of these COFs are water or moisture sensitive, restricting their device applications.

**Figure 6:**
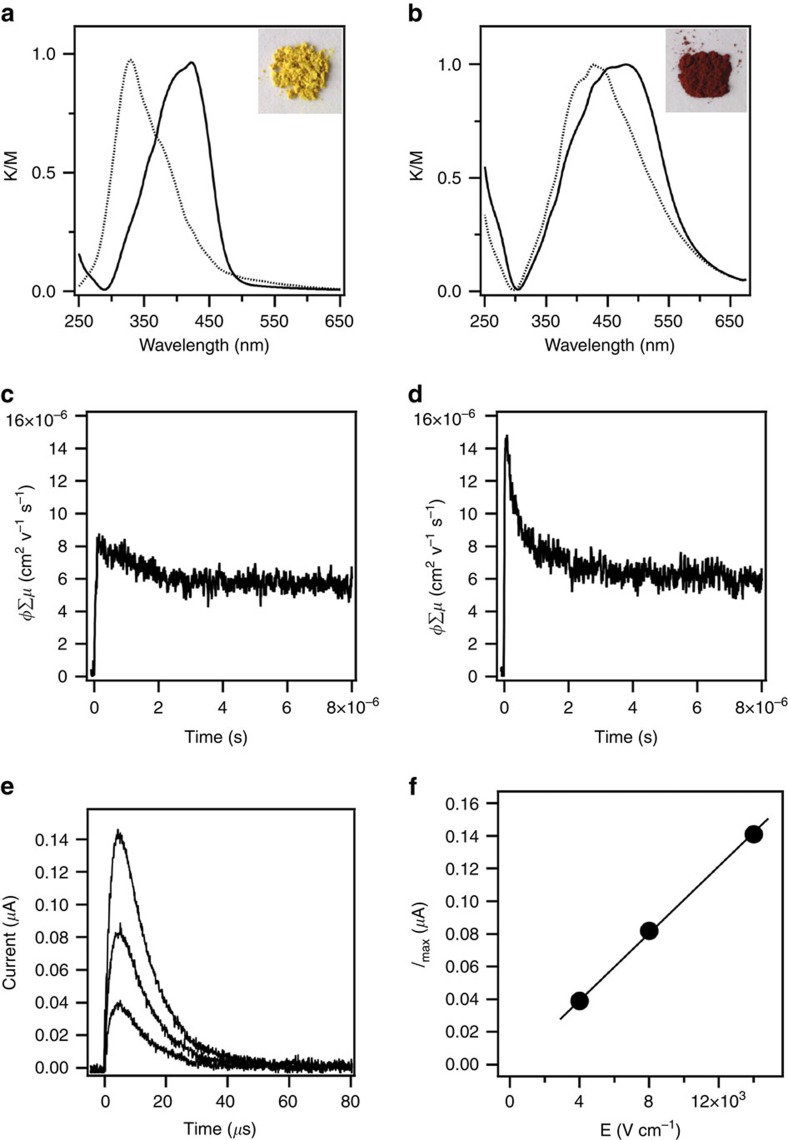
π-Electronic and conducting properties. (**a**) Solid-state electronic absorption spectra of HPB-COF (solid curve) and HPB (dotted curve). Inset: a photo of the HPB-COF sample. (**b**) Solid-state electronic absorption spectra of HBC-COF (solid curve) and HBC (dotted curve). Inset: a photo of the HBC-COF sample. (**c**) FP-TRMC profile of HPB-COF. (**d**) FP-TRMC profile of HBC-COF. (**e**) Photocurrent generation of spin coated HBC-COF on a comb-type gold electrode device (electrode gap=5 μm) at different bias voltages (2, 4 and 7 V). (**f**) *I*–*V* curve of HBC-COF on the comb-type gold electrode device.

Electronic absorption spectroscopy was conducted to evaluate the π-electronic functions. Compared with the HPB monomer that exhibits an absorption band at 329 nm, HPB-COF (yellow colour, Fig. 6a inset) exhibited a 94 nm redshifted absorption band centred at 423 nm (Fig. 6a). By contrast, HBC-COF (deep red colour, Fig. 6b inset) exhibited an absorption band at 480 nm (Fig. 6b), which is redshifted by 55 nm from that of the HBC monomer (425 nm). Therefore, the triangular COFs constitute 2D conjugated polymer networks, which allow for extended π-cloud delocalization over the ordered frameworks.

To obtain insight into the electronic properties of HPB-COF and HBC-COF, we evaluated the highest occupied molecular orbital (HOMO) and the lowest unoccupied molecular orbital (LUMO) energy gaps using DFTB calculations (Supplementary Tables 3 and 4). For HPB-COF, the monolayer has a HOMO-LUMO gap of 2.30 eV, whereas upon eclipsed hybrid AA stacking the gap decreased to 2.17 eV. The monolayer of HBC-COF has a small gap of 2.00 eV, which is further decreased to 1.71 eV for the 0.8 Å slipped AA stacking structure (Table 1). A low energy gap facilitates charge carrier transport in the frameworks; therefore, HBC-COF is superior to HPB-COF in charge carrier transport as evidenced by its low energy gaps. The triangular topology allows for intra-sheet π-cloud delocalization, whereas the π-columns of *C*_6_-symmetric vertices further promote inter-sheet π-cloud delocalization. Frontier orbital mapping suggests that the HOMO is centred on the HPB and HBC vertices, whereas the LUMO is located on the phenyl edges (Supplementary Fig. 22).

We further investigated the photoconductivity of HPB-COF and HBC-COF using the flash photolysis time-resolved microwave conductivity (FP-TRMC) method^41^,^42^. Upon excitation with a 355-nm laser pulse at a photon density of 9 × 10^15^ photons cm^−2^, HPB-COF under air yielded a *ϕ*Σ*μ* value (*ϕ*: photocarrier generation quantum yield, Σ*μ*: the sum of charge carrier mobility) of 0.8 × 10^−5^ cm^2^ V^−1^ s^−1^ (Fig. 6c). By contrast, under otherwise identical conditions, HBC-COF exhibited a *ϕ*Σ*μ* value of 1.5 × 10^−5^ cm^2^ V^−1^ s^−1^ (Fig. 6d), which is twice that of HPB-COF. The *ϕ*Σ*μ* value of HBC-COF is one to two orders of magnitude higher than those of representative conducting tetrathiafulvalene COFs^26^. After the initial time, a decay for both cases can be assumed due to charge re-combinations, whereas the retained tails suggest the presence of long-lived charges in the COFs.

To evaluate the hole mobility of HBC-COF, the *ϕ* value was determined by the direct current integration method^42^,^43^,^44^. The HBC-COF on comb-type electrode devices was exposed to a 355-nm laser pulse at 9.1 × 10^15^ photons cm^−2^, and the time-of-flight transient photocurrent was monitored at different bias voltages (Fig. 6e). A linear *I*–*V* curve indicates ohmic behaviour for the electric conduction of HBC-COF (Fig. 6f). By comparing the photocurrents with those of poly(9,9'-dioctylfluorene)^42^, the *ϕ* value of HBC-COF was estimated to be 2.1 × 10^−5^. The combination of these experiments allows for the calculation of an intrinsic hole mobility as high as 0.7 cm^2^ V^−1^ s^−1^. Notably, this hole mobility is 40–70-fold higher than those of triphenylene-based COF (0.01 cm^2^ V^−1^ s^−1^)^32^ and porphyrin-knoted ZnP-COF (0.016 cm^2^ V^−1^ s^−1^)^25^, and is among the highest reported for 2D COFs^10^,^21^,^22^,^24^. These results indicate that the triangular yet stable COFs can be used in semiconducting devices.

In summary, we have developed a triangular topology for the design and synthesis of an unprecedented class of COFs. From a porous material point of view, the triangular topology provides a straightforward method of preparing microporous 2D COFs. From a π-electronic perspective, the triangular topology enables the periodic ordering of π-columnar arrays at the highest density. As demonstrated by HPB-COF and HBC-COF, the triangular COFs offer crystallinity, microporosity, stability, π-cloud delocalization and photoconductivity; the materials with these multifunctions may find a wide variety of applications, such as gas storage, size-selective separation, catalysis and the manufacture of sensing, semiconducting and optoelectronic devices. Thus, this study opens a new chemical approach to the structural and functional design of crystalline porous organic materials.

## Methods

### Synthesis of HPB-COF

A 10-ml Pyrex tube was charged with [NH_2_]_6_HPB (10.0 mg, 0.0094, mmol), toluene (2 ml), terephthaldehyde (6.44 mg, 0.05 mmol) and 0.1 ml AcOH (3 M). The mixture was degassed through three freeze–pump–thaw cycles, sealed under vacuum and heated at 120 °C for 9 days. The mixture was cooled to room temperature and the yellow precipitate was collected by centrifugation, washed with tetrahydrofuran several times, and dried under vacuum for 12 h to afford HPB-COF in a 70% isolated yield. Other reaction conditions (Supplementary Table 1, Supplementary Figs 10 and 12), such as toluene/6 M AcOH (20/1 v/v), toluene/dioxane/3 M AcOH (19/1/1 v/v) and toluene/dioxane/6 M AcOH (19/1/1 v/v), followed the same experimental procedure.

### Synthesis of HBC-COF

A 10-ml Pyrex tube was charged with [NH_2_]_6_HBC (10.0 mg, 0.0094, mmol), dioxane/*n*-butanol (2 ml, 19/1 v/v), terephthaldehyde (3.76 mg, 0.028 mmol) and 0.1 ml AcOH (6 M). The mixture was degassed through three freeze–pump–thaw cycles, sealed under vacuum and heated at 120 °C for 12 days. The reaction mixture was cooled to room temperature and the deep red precipitate was collected by centrifugation, washed with THF several times, and dried under vacuum for 12 h to afford HBC-COF in a 95% isolated yield. Other solvent conditions (Supplementary Table 2, Supplementary Figs 11 and 12), such as toluene/dioxane/6 M AcOH (1/19/1 v/v), mesitylene/dioxane/6 M AcOH (1/19/1 v/v), mesitylene/THF/6 M AcOH (15/5/1 v/v) and *o*-DCB/THF/6 M AcOH (15/5/1 v/v), followed the same experimental procedure.

### FP-TRMC

The charge carrier mobility was measured by the FP-TRMC technique at 20 °C under air. Film samples on a quartz plate were prepared by drop-casting a toluene solution that contains HPB-COF or HBC-COF together with polystyrene. Charge carriers were photochemically generated using the third harmonic generation (*λ*=355 nm) of a Spectra Physics model INDI-HG Nd:YAG laser with a pulse duration of 5–8 ns. The photon density of the 355-nm pulse was 9.0 × 10^15^ photons cm^−2^. The microwave frequency and power were set at ∼9.1 GHz and 3 mW, respectively. The TRMC signal, picked up by a diode (rise time<1 ns), was monitored with a Tektronics model TDS3052B digital oscilloscope. The observed conductivities were normalized, based on the photocarrier generation yield (*ϕ*) multiplied by sum of the charge carrier mobilities (Σ*μ*), according to the equation, *ϕ*Σ*μ*=(1/*e*A*I*_0_*F*_light_)(*ΔP*_r_/*P*_r_), where *e*, A, *I*_0_, *F*_light_, *P*_r_ and *ΔP*_r_ are the unit charge of a single electron, sensitivity factor (S^−1^ cm^−1^), incident photon density of the excitation laser (photon cm^−2^), correction (or filling) factor (cm^−1^), and reflected microwave power and its change, respectively.

### Current integration method

The *ϕ* values were determined by the direct current integration method at 20 °C. An interdigitated comb-type gold electrode device with 5 μm gaps, 40 nm height and 2 mm width was fabricated by a lithographic process on a glass substrate. Samples were fabricated by spin-coating a toluene suspension of finely grounded HBC-COF on the electrodes. The samples were exposed to a 355-nm pulse with 9.0 × 10^15^ photons cm^−2^. Current transients were monitored by a Tektronix model TDS3052B digital oscilloscope equipped with a 10 kΩ termination resistance. The applied voltage was set at 2, 4 and 7 V by an Advantest model R8252 digital electrometer. Using a *ϕ* value of 2.3 × 10^−4^ for poly(9,9'-dioctylfluorene) film as a reference, the *ϕ* values of HBC-COF were estimated by comparing the photocurrent peaks with those of poly(9,9'-dioctylfluorene) under the same bias voltage.

## Additional information

**How to cite this article:** Dalapati, S. *et al*. Rational design of crystalline supermicroporous covalent organic frameworks with triangular topologies. *Nat. Commun*. 6:7786 doi: 10.1038/ncomms8786 (2015).

## Supplementary Material

Supplementary Information Supplementary Figures 1-22, Supplementary Tables 1-6, Supplementary Methods and Supplementary References

## Figures and Tables

**Table 1:** Unit cell structure and property.

**COFs**	***a*****=*****b*** **(Å)**	***c*** **(Å)**	**Total crystal stacking energy (kcal mol**^**–1**^)	**Pore size (nm)**	**π-Column density (nm**^**–2**^**)**	**Band gap (eV)**
HPB-COF	21.57	5.17	46.74	1.2	0.25	2.17
HBC-COF	30.14	3.54	136.37	1.8	0.13	1.71

COF, covalent organic framework; HBC-COF, hexabenzocoronene-COF; HPB-COF, hexaphenylbenzene-COF.
