# Supplementary material for: Rational design of crystalline supermicroporous covalent organic frameworks with triangular topologies
Source: Nat Commun. 2015 Jul 16;6:7786. doi: 10.1038/ncomms8786 (PMC4518282; doi:10.1038/ncomms8786)
Supplement: Supplementary Information — Supplementary Figures 1-22, Supplementary Tables 1-6, Supplementary Methods and Supplementary References [file ncomms8786-s1.pdf]

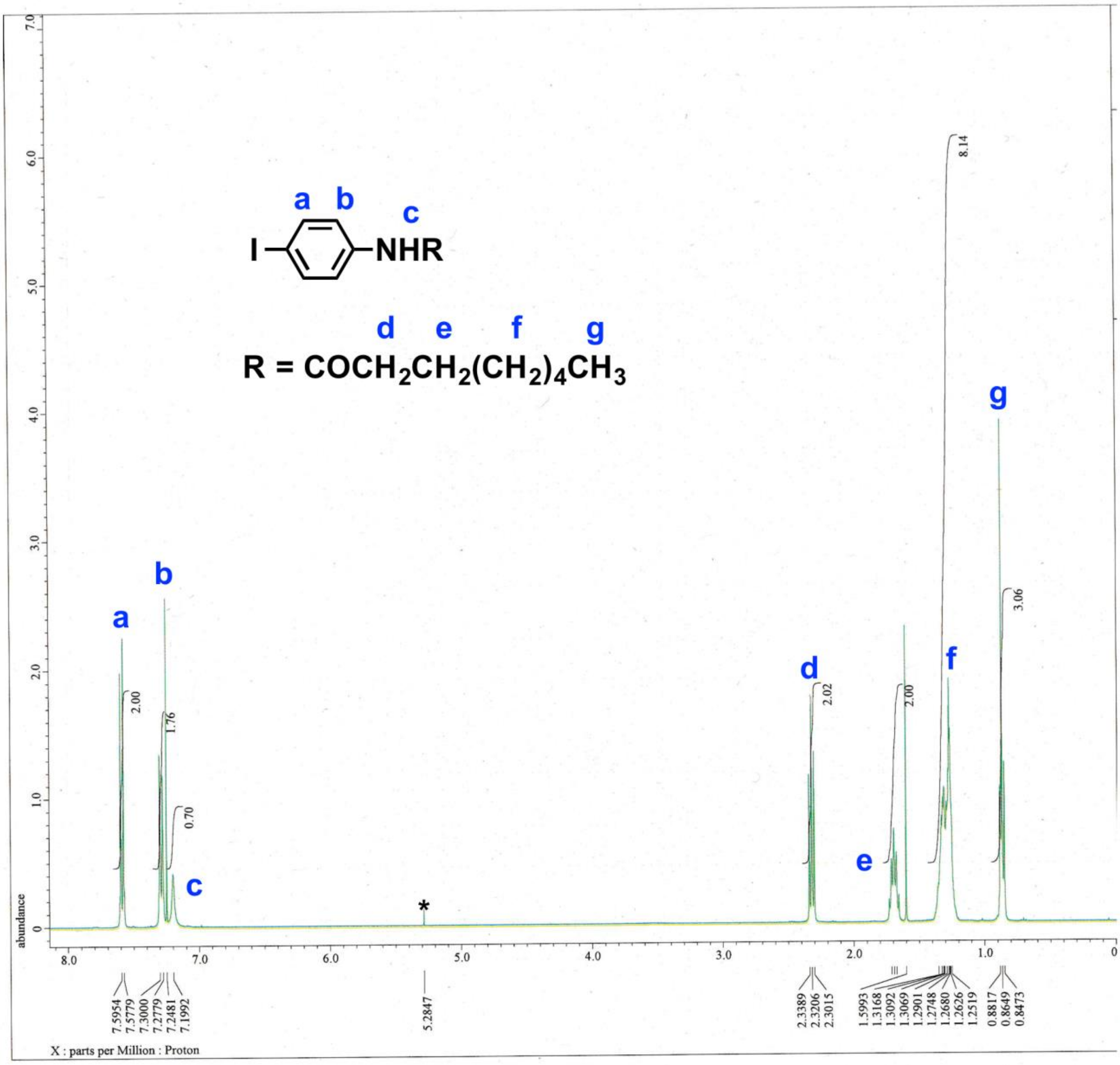

Supplementary Figure 1 | ^{1}H NMR spectra of octanoylamino-4-iodobenzene in $CDCl_3$ (* CH_2Cl_2).

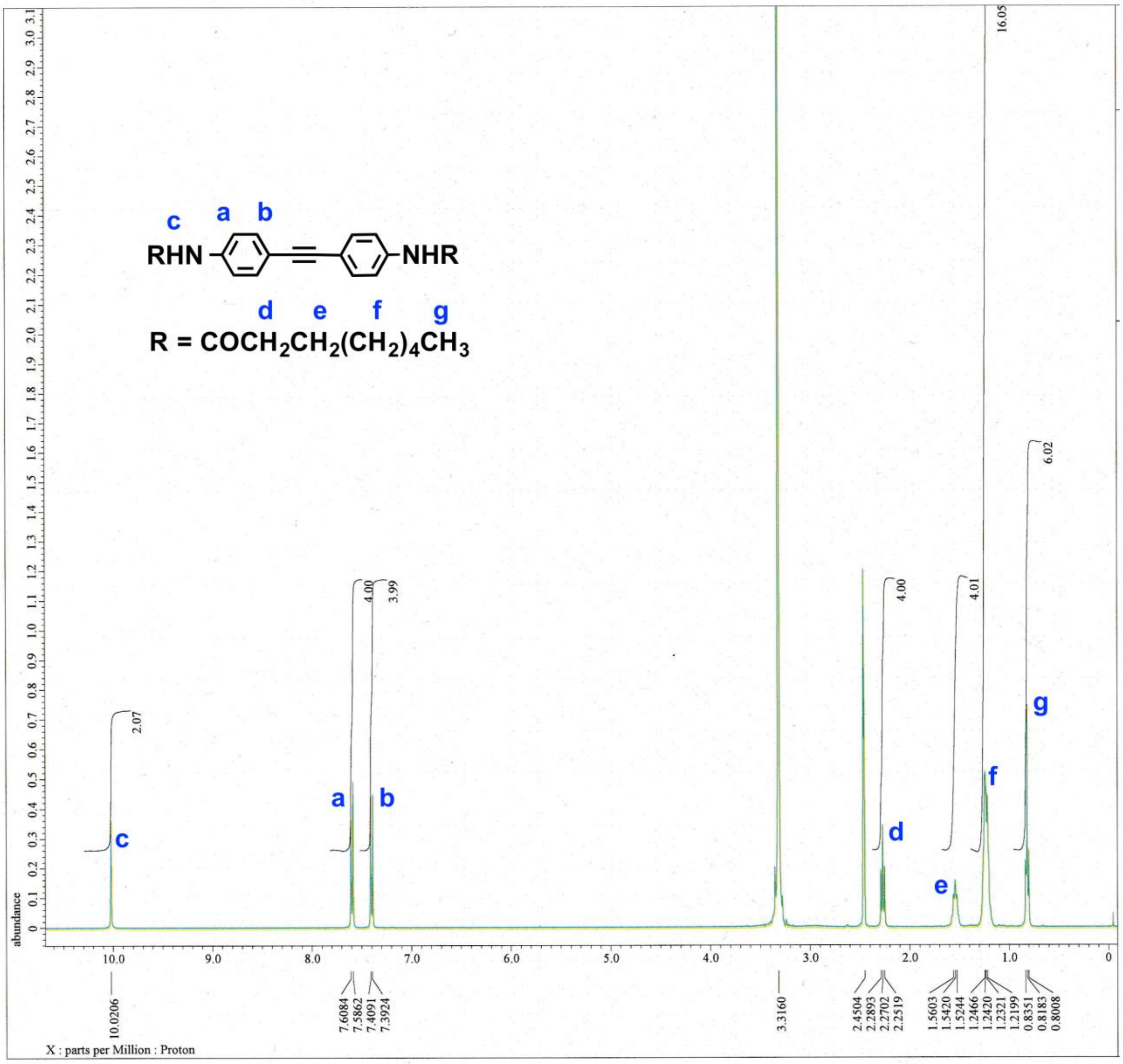

Supplementary Figure 2 | ^{1}H NMR spectra of bis[4-octanoylamino-phenyl]acetylene in d_6-DMSO.

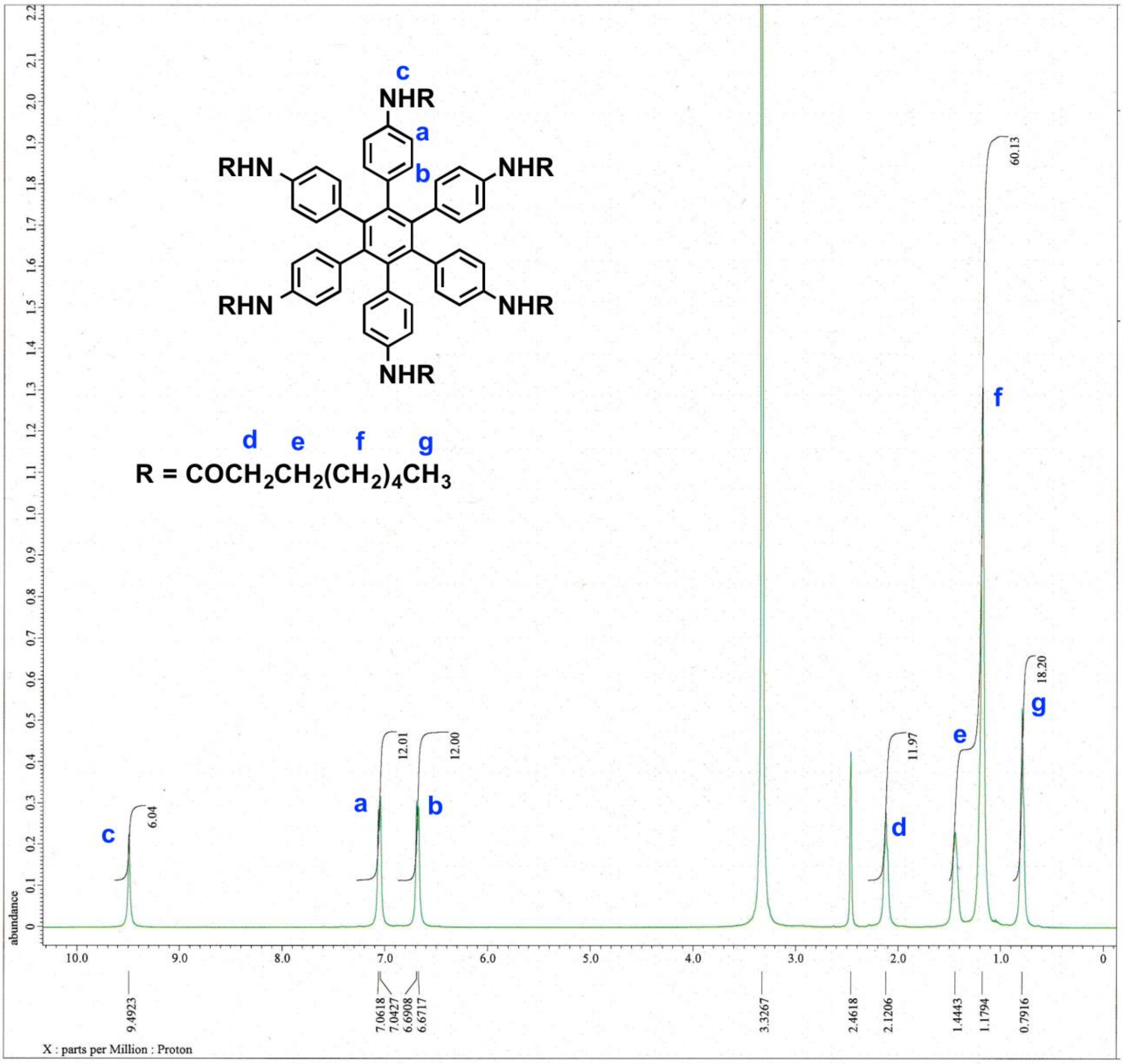

Supplementary Figure 3 | ^{1}H NMR spectra of $[RNH]_6$HPB in d_6-DMSO.

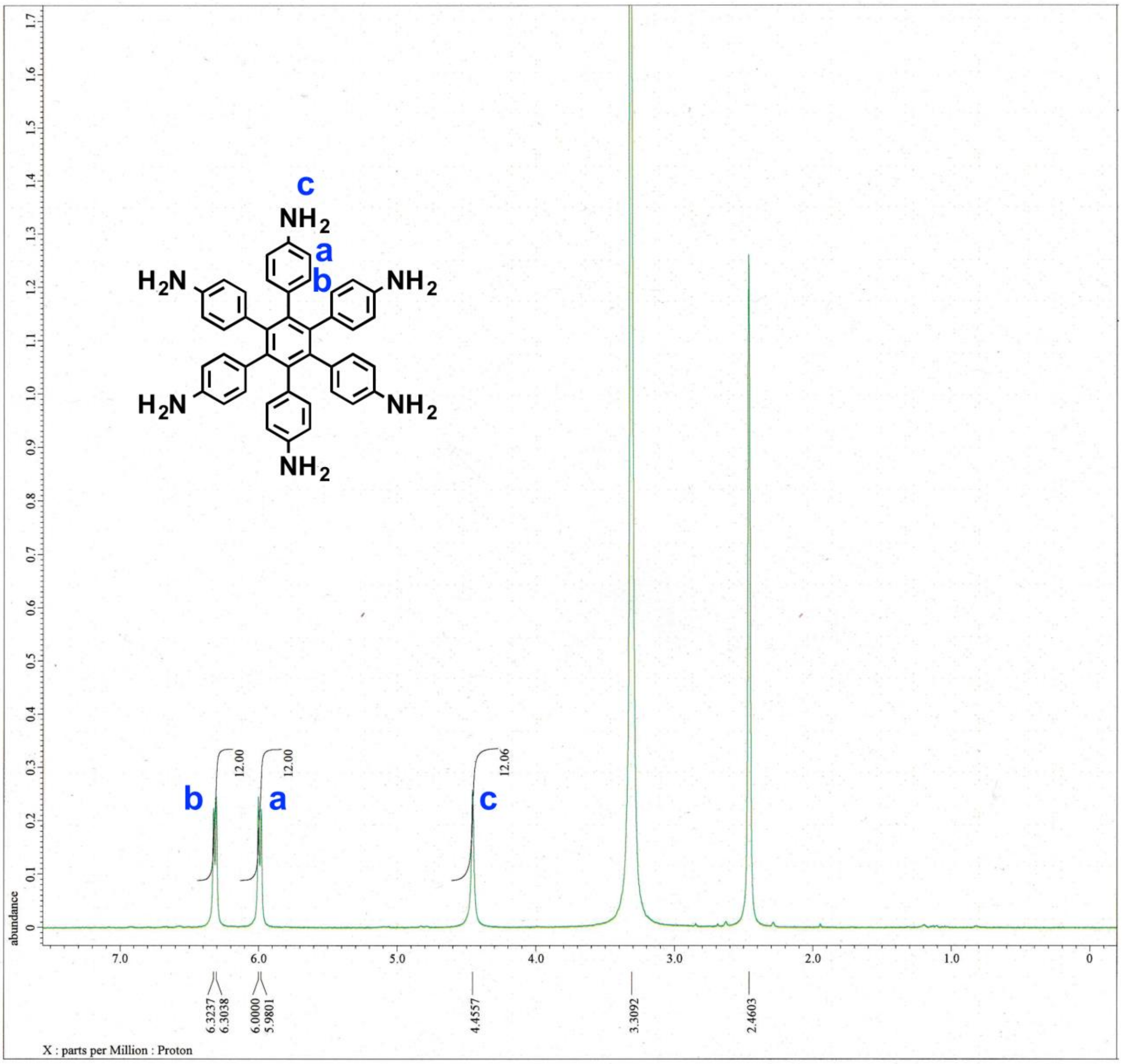

Supplementary Figure 4 | ^{1}H NMR spectra of $[NH_2]_6$HPB in d_6-DMSO.

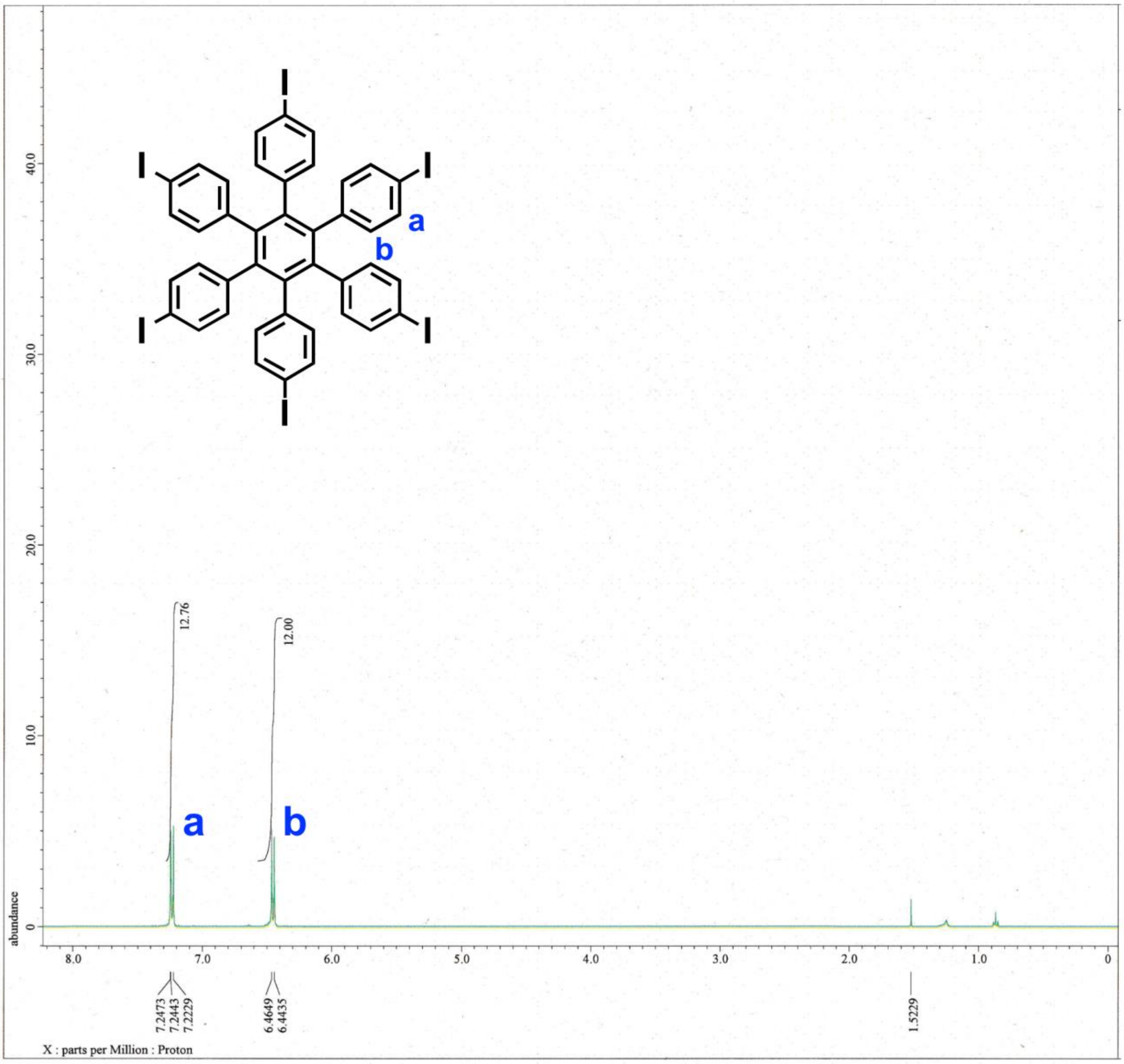

Supplementary Figure 5 | ^{1}H NMR spectra of $HPBI_6$ in $CDCl_3$.

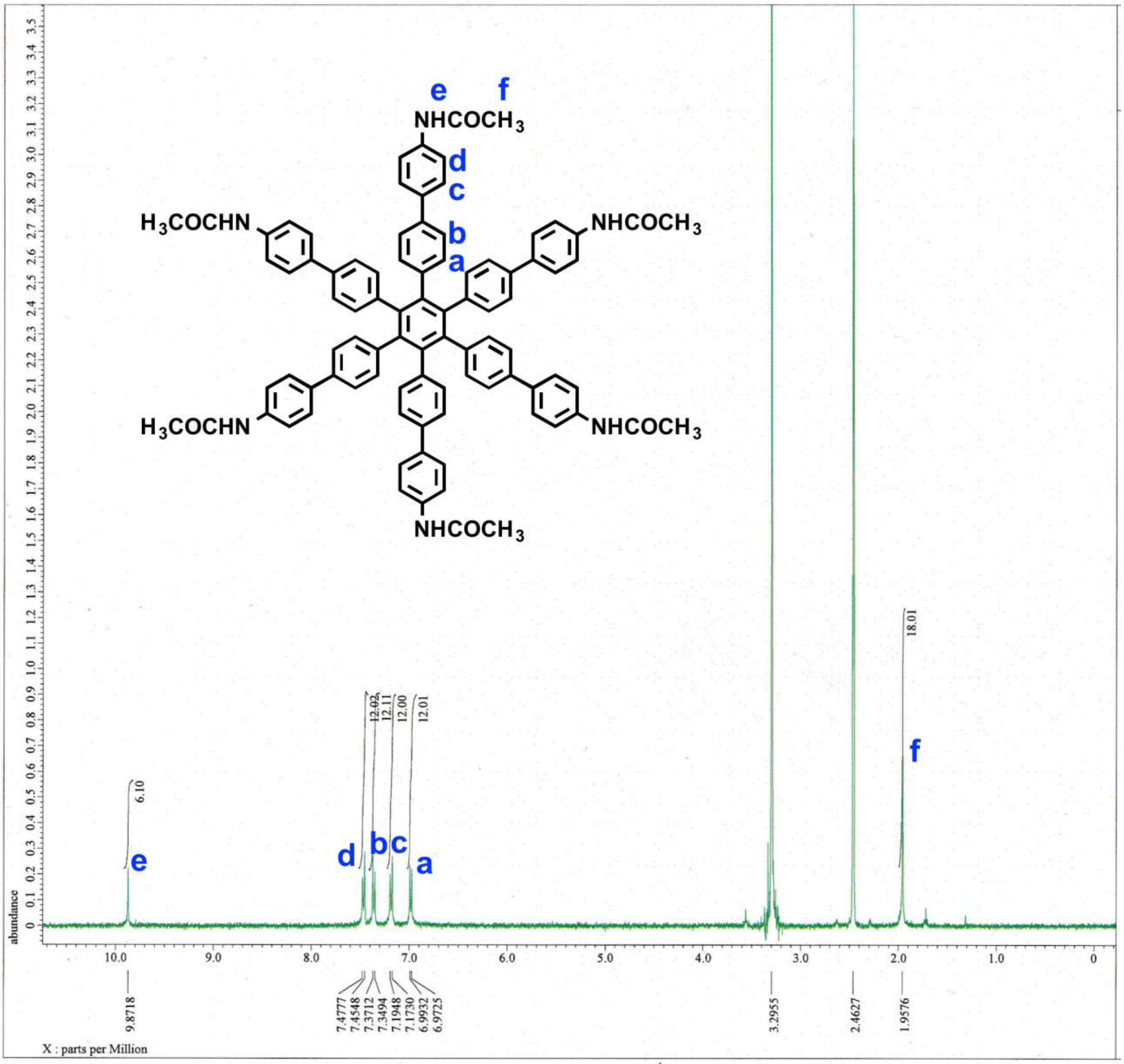

Supplementary Figure 6 | ^{1}H NMR spectra of $[CH_3CONH\text{-}HP]_6HPB$ in d_6-DMSO.

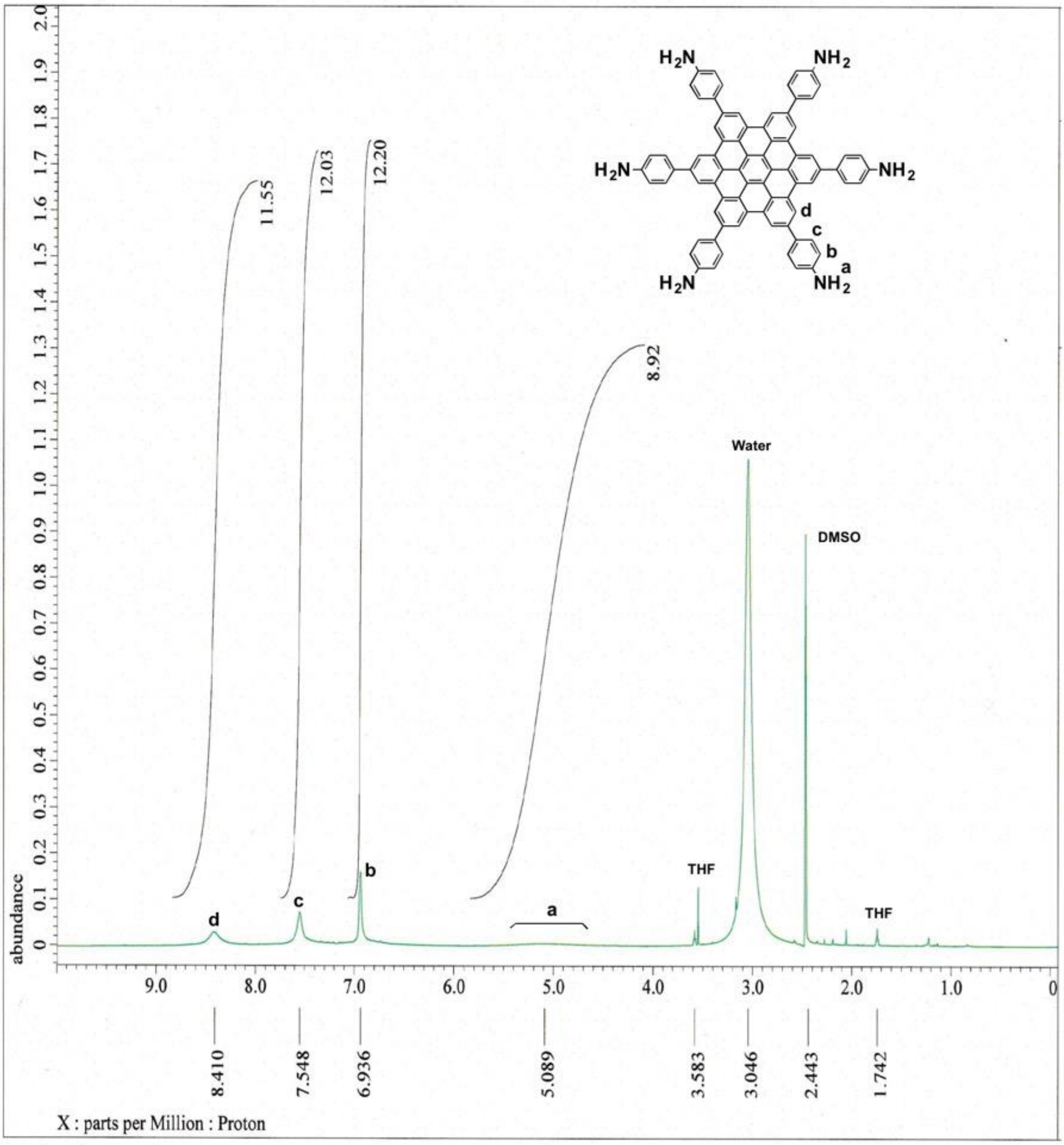

Supplementary Figure 7 | ^{1}H NMR spectra of $[NH_2]_6$HBC in d_6-DMSO. A suspension of $[NH_2]_6$HBC (20 mg) in d_6-DMSO (1 mL) was degassed via three freeze-pump-thaw cycles, sealed and put in oven (120 °C) for 5 h, in order to dissolve the monomer. The solid part was removed by using centrifuge (4000 rpm, 2 min) and the upper solution was subjected to ^{1}HNMR spectroscopy at 80 °C. The ratio of the proton numbers was a:b:c:d = 9:12:12:12, which was consistent with the structure (12:12:12:12); the NH_2 groups may cause proton exchanges with D in this case, thus decreasing the NH_2 proton integration. Owing to the big hexabenzocoronene (HBC) unit in the core, the protons were shifted to lower fields, compared to those of $[NH_2]_6$HPB.

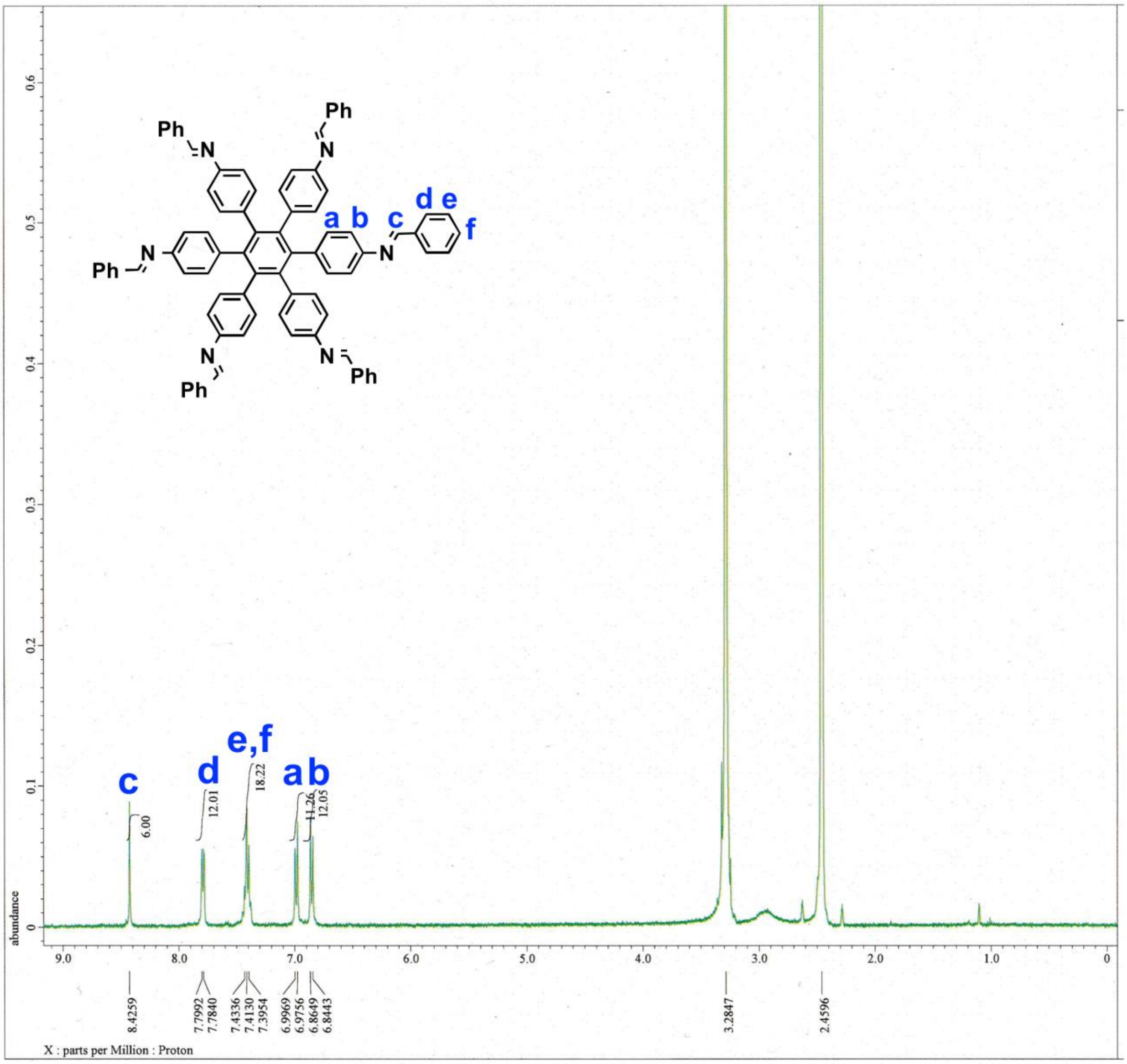

Supplementary Figure 8 | ^{1}H NMR spectra of HPB-Ph in d_6-DMSO.

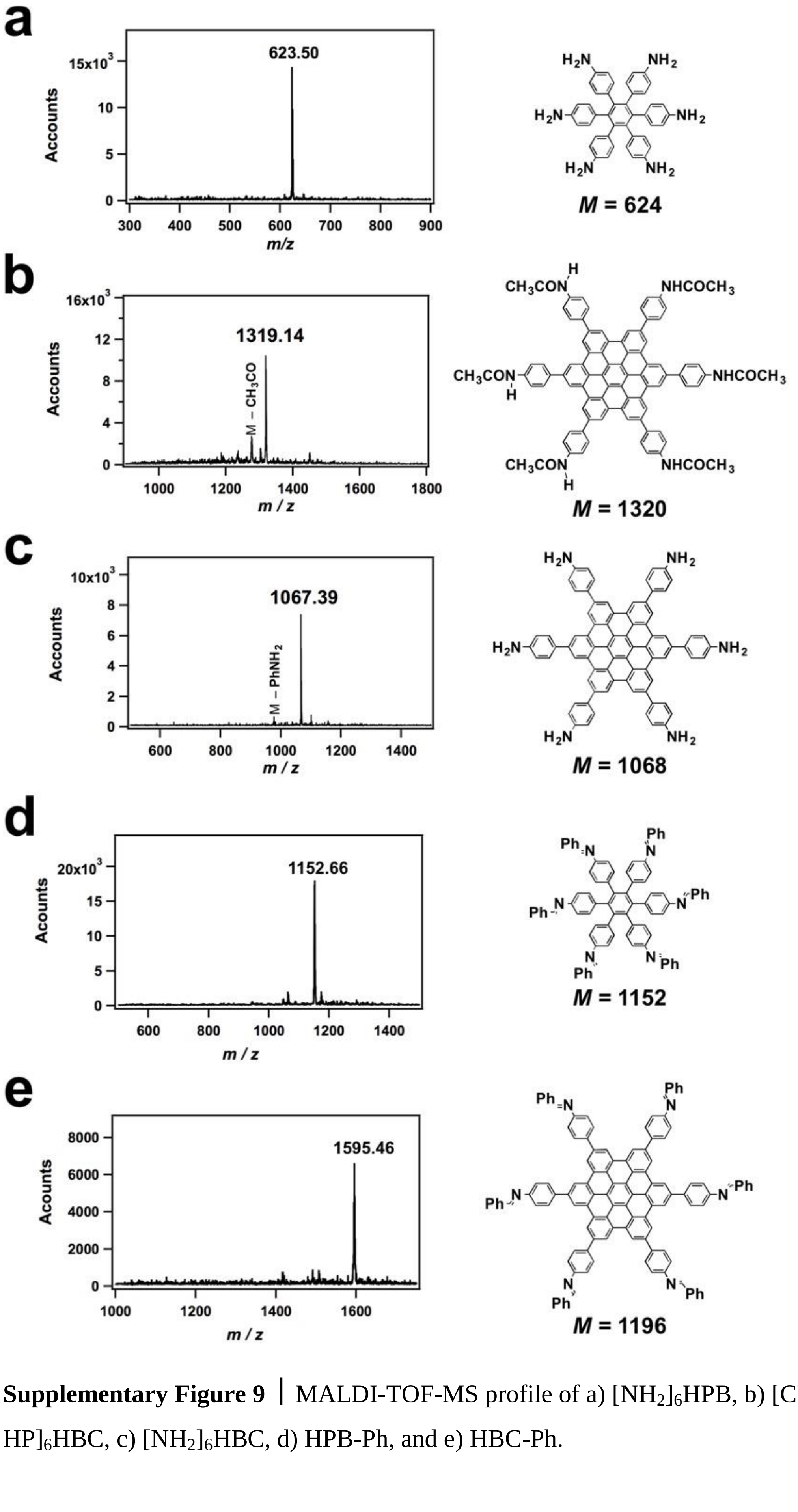

Supplementary Figure 9 | MALDI-TOF-MS profile of a) $[NH_2]_6$HPB, b) $[CH_3CONH$-$HP]_6$HBC, c) $[NH_2]_6$HBC, d) HPB-Ph, and e) HBC-Ph.

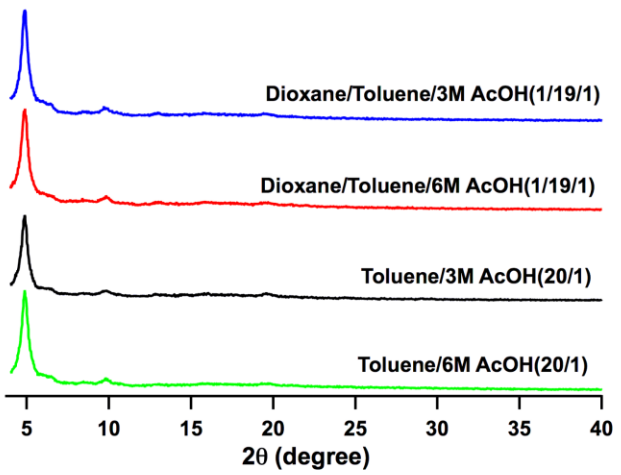

Supplementary Figure 10 | XRD profiles of HPB-COF synthesized under different conditions.

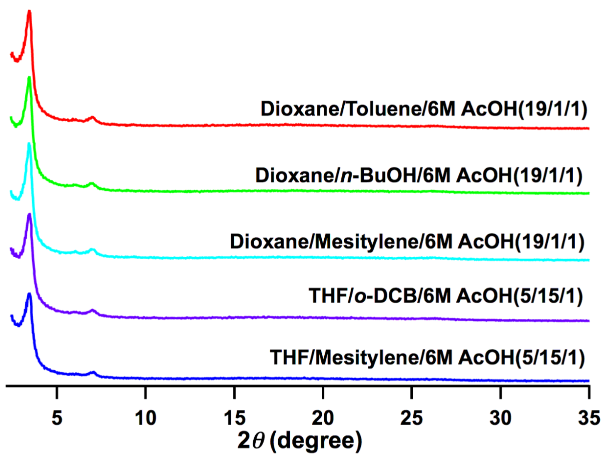

Supplementary Figure 11 | XRD profiles of HBC-COF synthesized under different conditions.

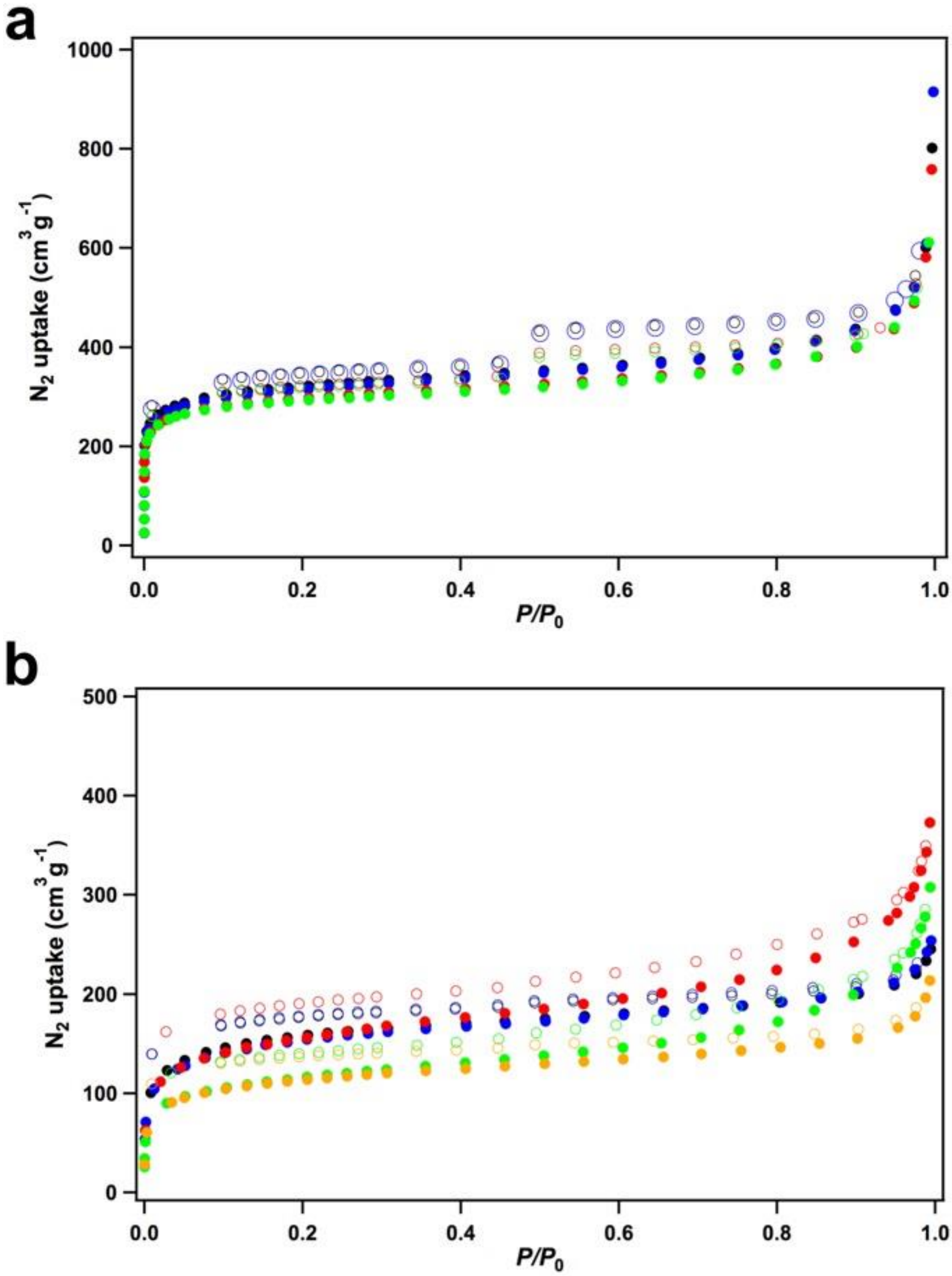

Supplementary Figure 12 | Nitrogen sorption isotherm curves of a) HPB-COF (black: toluene/3 M AcOH; blue: dioxane/toluene (1/19)/ 3 M AcOH; red: toluene/6 M AcOH; green: dioxane/toluene (1/19)/ 6 M AcOH) and b) HBC-COF (black: dioxane/*n*-butanol (19/1)/6 M AcOH; blue: dioxane/toluene (19/1)/6 M AcOH; red: dioxane/mesitylene (19/1)/6 M AcOH; green: THF/mesitylene (5/15)/6 M AcOH; orange: THF/*o*-DCB (5/15)/6 M AcOH;) synthesized in different solvents. Open circles are for desorption and filled circles are for adsorption.

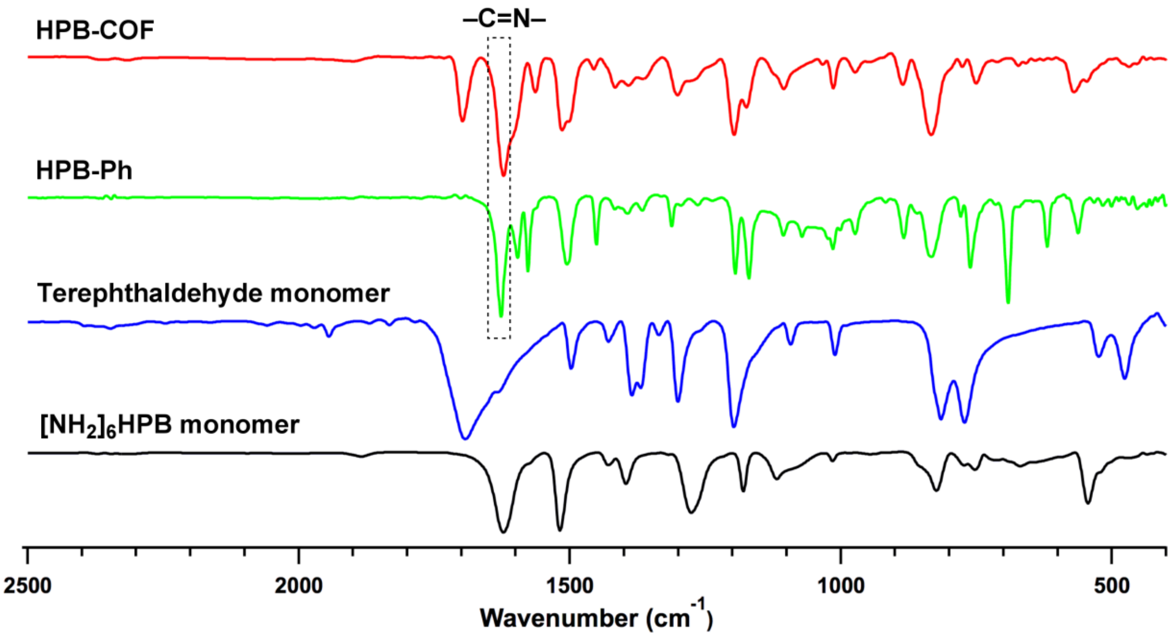

**Supplementary Figure 13 | ** FT-IR spectra of HPB monomer (black curve), terephthalaldehyde monomer (blue curve), HPB-Ph (green curve), and HPB-COF (red curve).

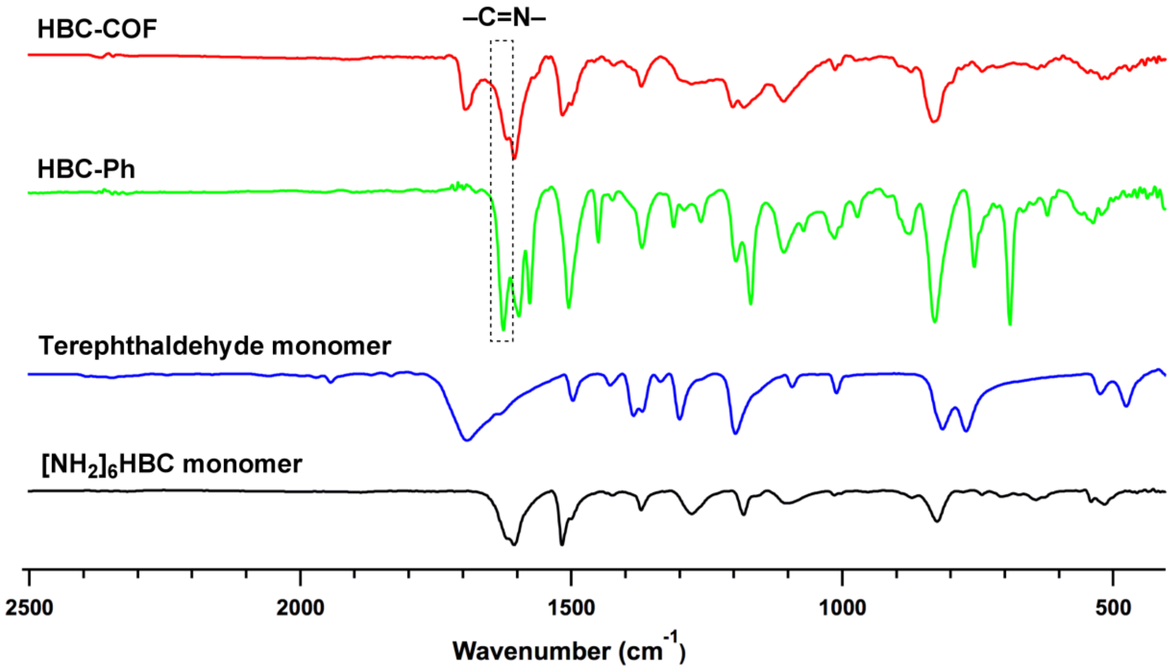

**Supplementary Figure 14 | ** FT-IR spectra of HBC monomer (black curve), terephthalaldehyde monomer (blue curve), HBC-Ph (green curve), and HBC-COF (red curve).

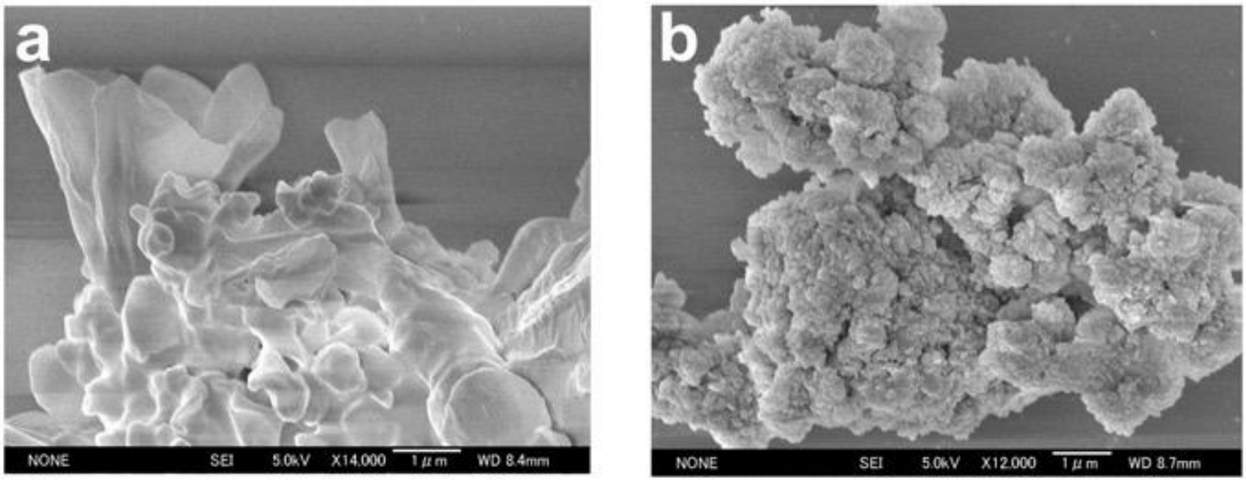

Supplementary Figure 15 | FE-SEM images of a) HPB-COF and b) HBC-COF.

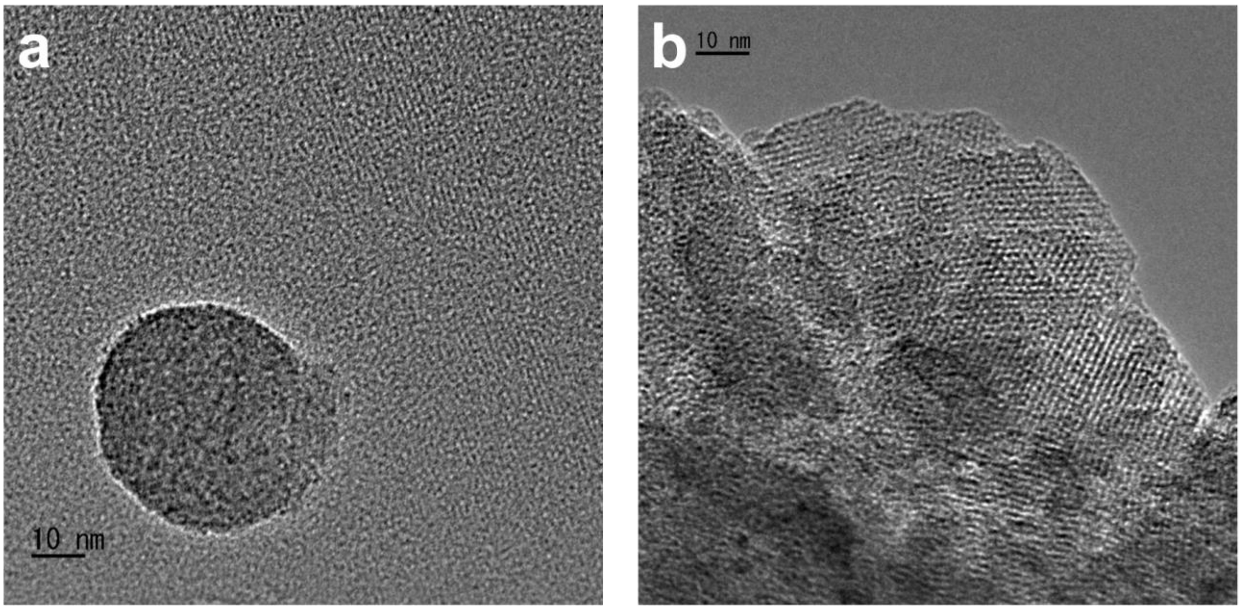

Supplementary Figure 16 | HR-TEM images of a) HPB-COF and b) HBC-COF.

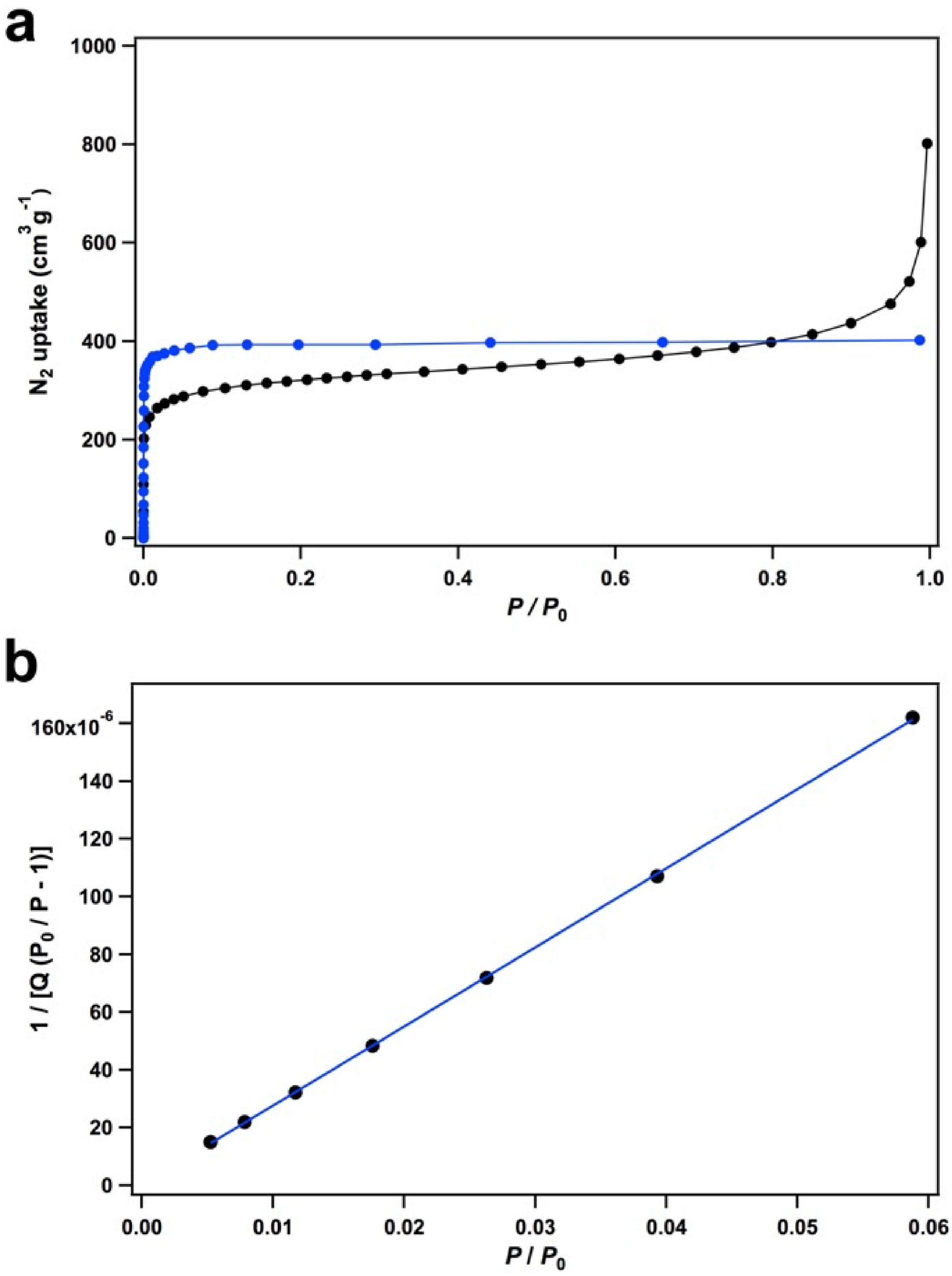

Supplementary Figure 17 | (a) Nitrogen sorption isotherm curves of HPB-COF at 77 K (black: experimentally measured curve; blue: theoretically calculated curve). (b) Simulated BET surface area plot of HPB-COF at 77 K using nitrogen as adsorbate. The V_m = 364.9 cm^3 g^{-1} (STP) and coefficient of determination R^2 = 0.9999.

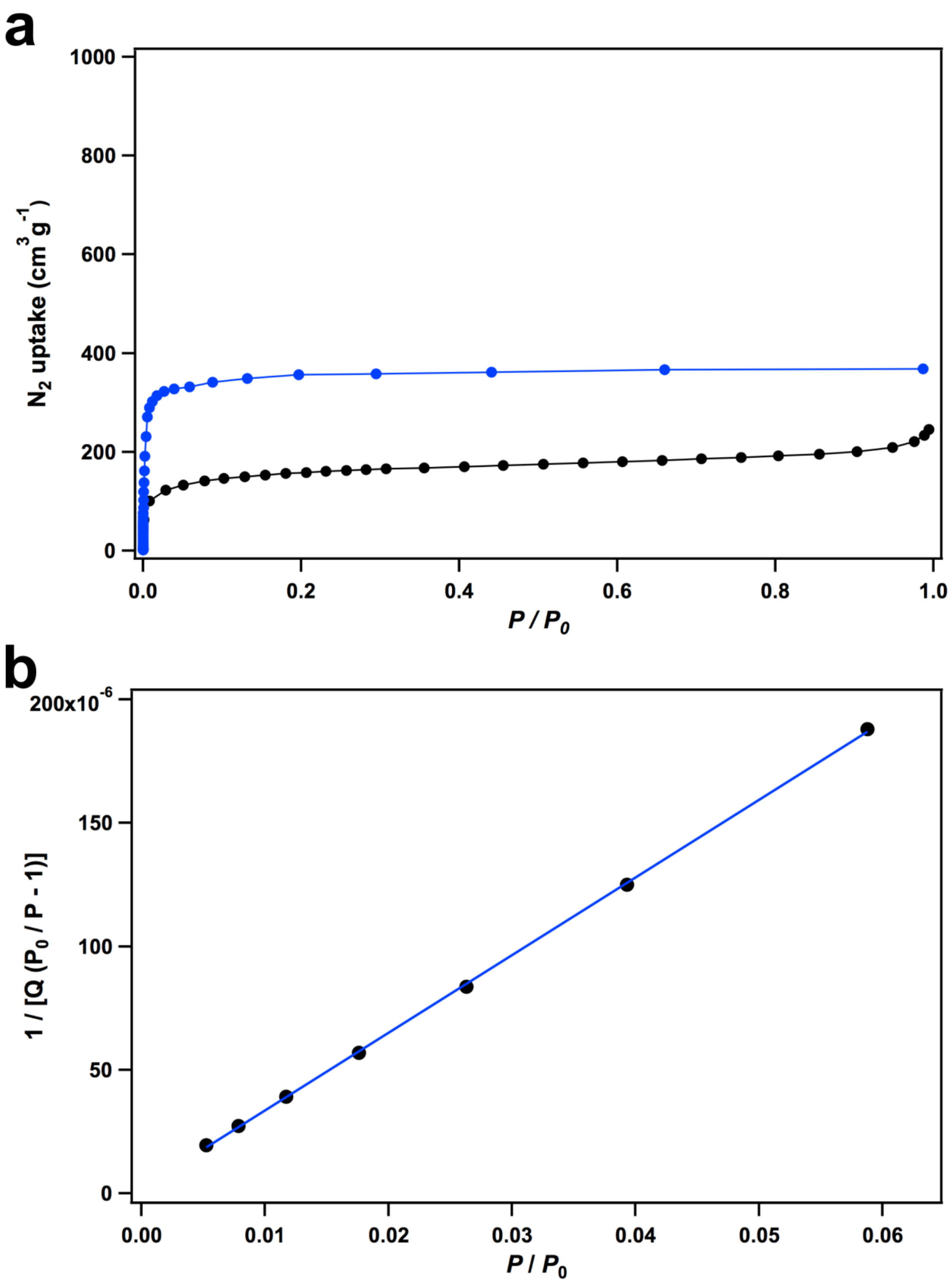

Supplementary Figure 18 | (a) Nitrogen sorption isotherm curves of HBC-COF at 77 K (black: experimentally measured curve; blue: theoretically calculated curve). (b) Simulated BET surface area plot of HBC-COF at 77 K using nitrogen as adsorbate. The V_m = 317.2 cm^3 g^{-1} (STP) and coefficient of determination R^2 =0.9998.

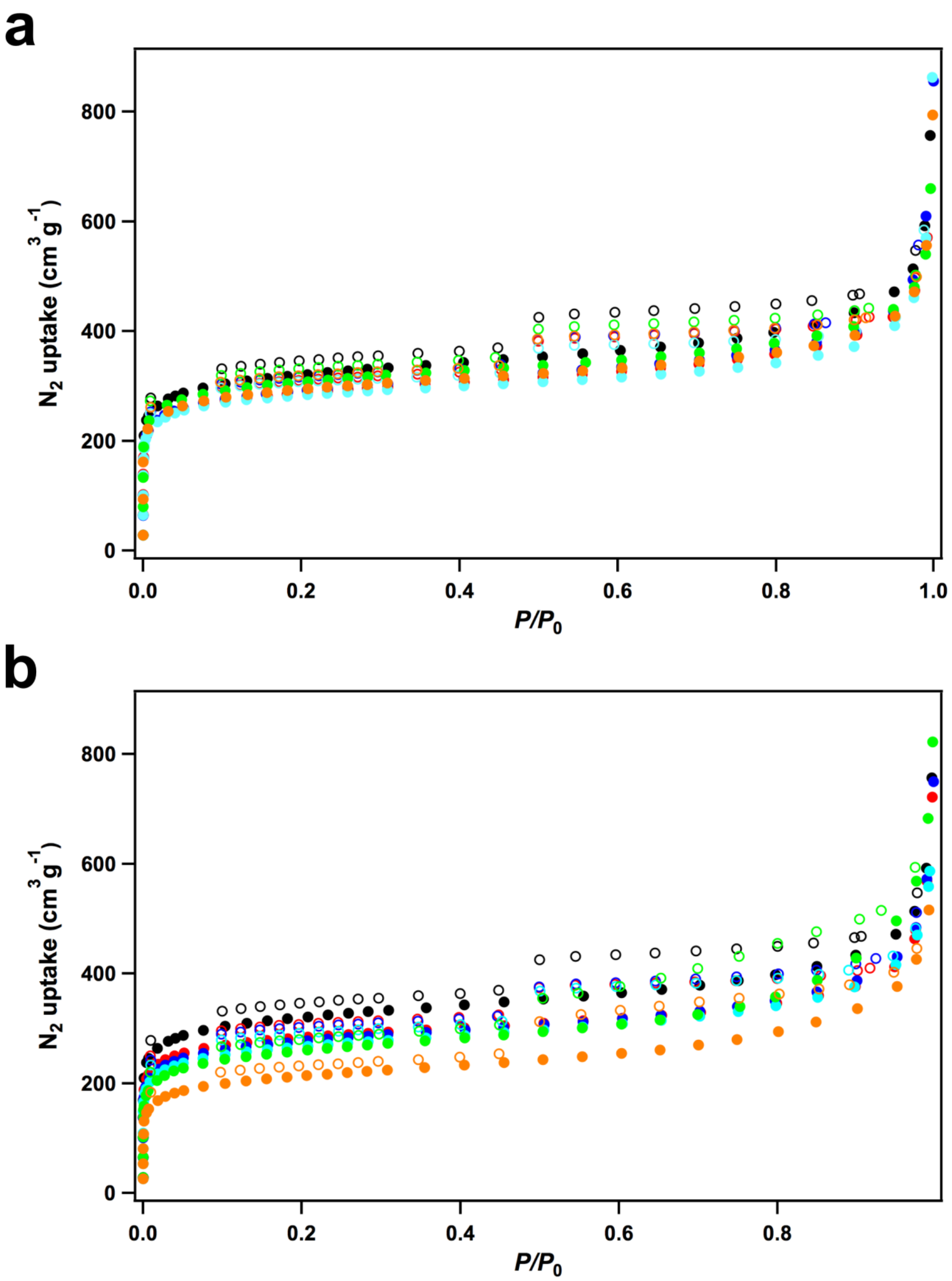

Supplementary Figure 19 | Nitrogen sorption isotherm curves of HPB-COF after treated at (a) 25 °C and (b) 100 °C for 1 day under different conditions (black: as synthesized; red: hexane; blue: THF; sky blue: methanol; green: water; orange: NaOH (1 M)). Open circles are for desorption and filled circles are for adsorption.

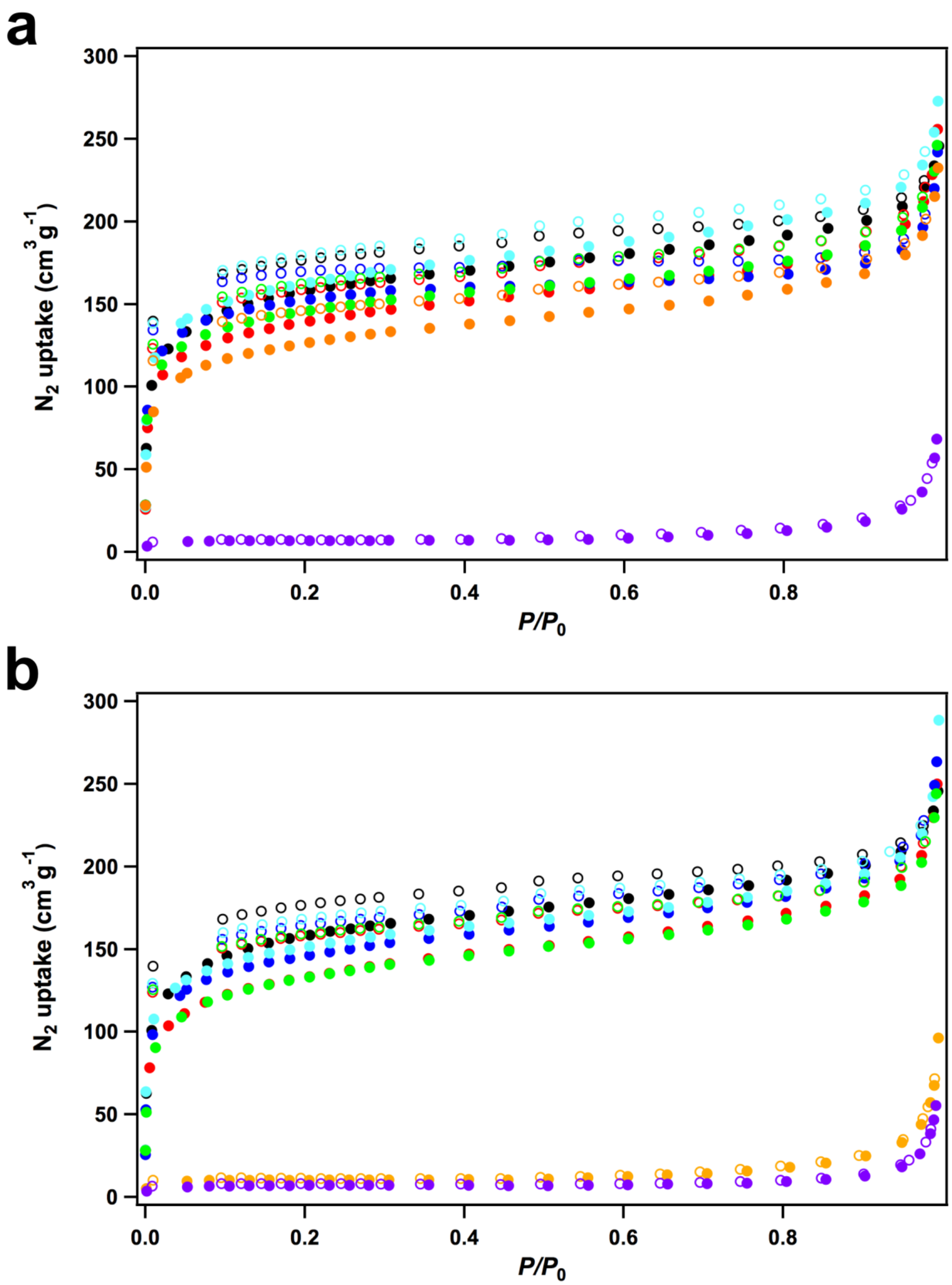

**Supplementary Figure 20 | ** Nitrogen sorption isotherm curves of HBC-COF after treated at (a) 25 °C and (b) 100 °C for 1 day under different conditions (black: as synthesized; red: hexane; blue: THF; sky blue: methanol; green: water; orange: NaOH (1 M); purple: HCl (1 M)). Open circles are for desorption and filled circles are for adsorption.

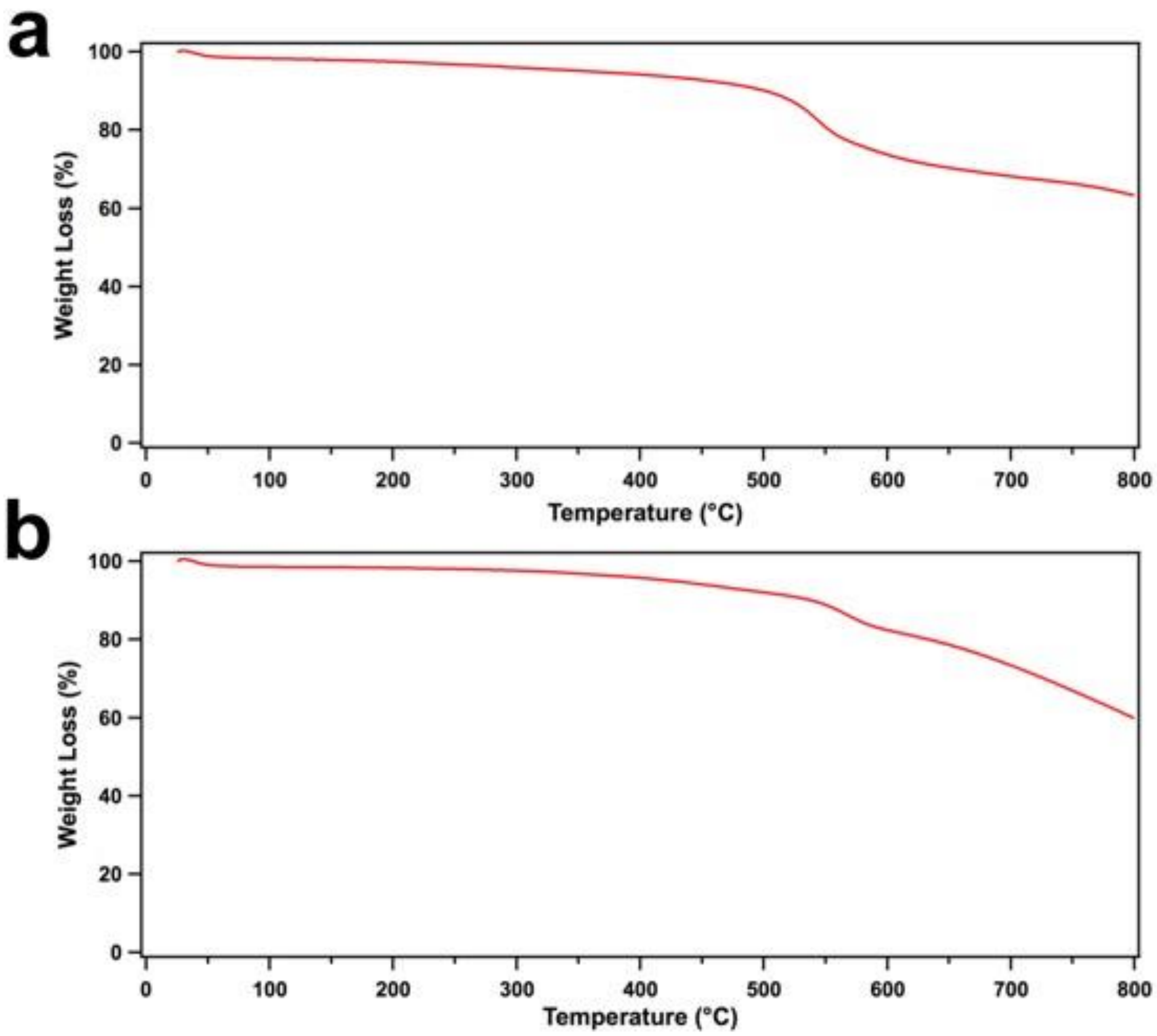

Supplementary Figure 21 | TGA profiles of a) HPB-COF and b) HBC-COF.

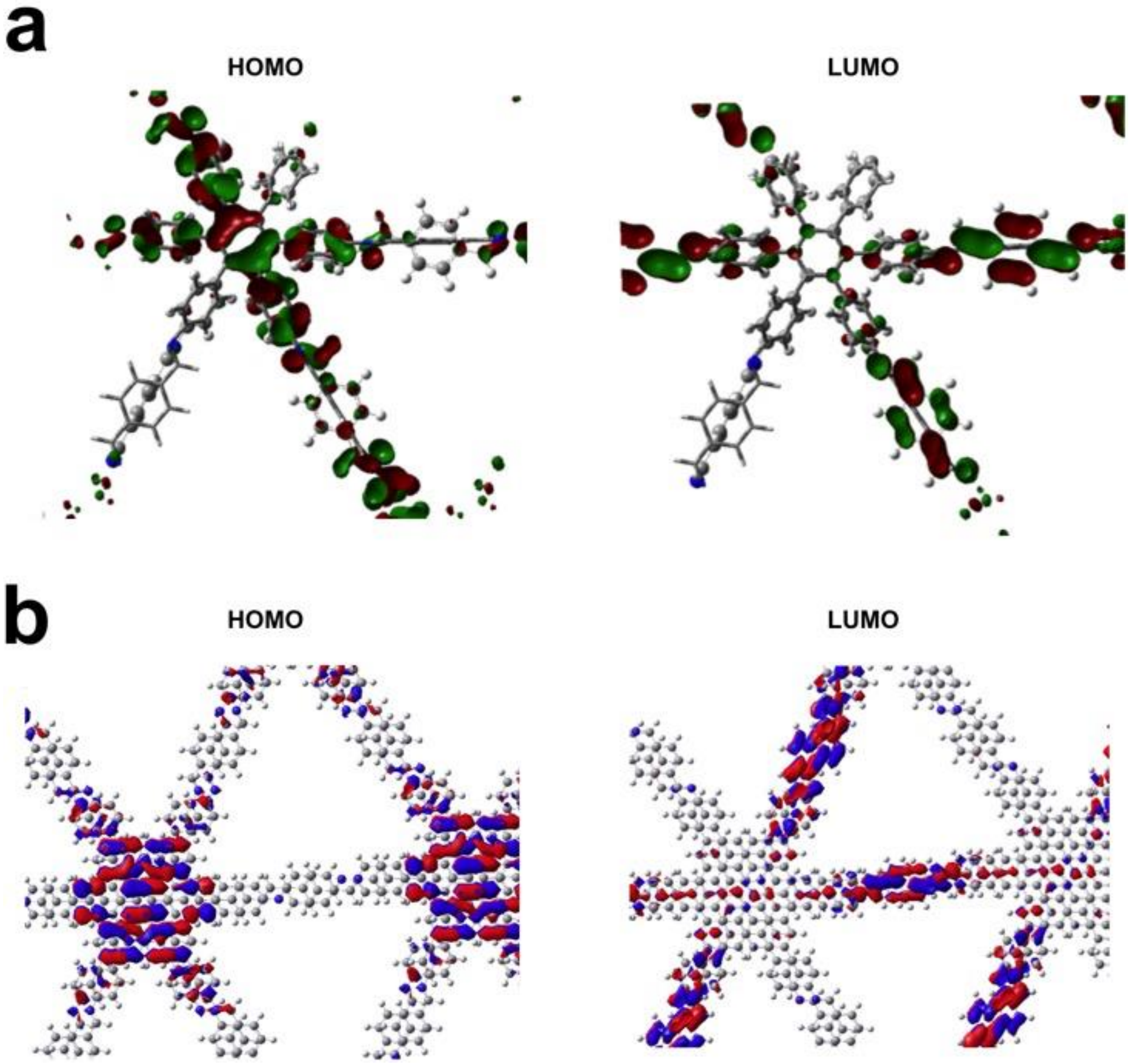

Supplementary Figure 22 | HOMO and LUMO mappings of a) HPB-COF and b) HBC-COF.

Supplementary Table 1 | Synthesis of HPB-COF under variable solvothermal conditions

Conditions	Temperature (°C)	Reaction Time (day)	BET Surface Area (m^2 g^{-1})	Pore Volume (cm^3 g^{-1})
Toluene/3M AcOH (20/1 by vol.)	120	9	965	0.79
Toluene/dioxane/3M AcOH (19/1/1 by vol.)	120	9	954	0.77
Toluene/6M AcOH (20/1 by vol.)	120	9	902	0.76
Toluene/dioxane/6M AcOH (19/1/1 by vol.)	120	9	896	0.74

Supplementary Table 2 | Synthesis of HBC-COF under variable solvothermal conditions

Conditions	Temperature (°C)	Reaction Time (day)	BET Surface Area (m^2 g^{-1})	Pore Volume (cm^3 g^{-1})
Toluene/dioxane/6M AcOH (1/19/1 by vol.)	120	12	459	0.43
Mesitylene/dioxane/6M AcOH (1/19/1 by vol.)	120	12	454	0.30
n–BuOH/dioxane/6M AcOH (1/19/1 by vol.)	120	12	469	0.29
Mesitylene/THF/6M AcOH (15/5/1 by vol.)	120	12	334	0.36
o–DCB/THF/6M AcOH (15/5/1 by vol.)	120	12	332	0.25

Supplementary Table 3 | The total DFTB energies, Lennard-Jones contributions (LJ), and the crystal stacking energies per monolayer as well as the corresponding HOMO-LUMO energy gap for HPB-COF

Stacking	*c* (Å)	Total DFTB Energy (a.u.)	LJ energy (a.u.)	Per unit crystal stacking energy (kcal mol^{-1})	HOMO-LUMO gap (eV)
Monolayer		–142.985866	0.5969	–	2.303
AA	5.16	–286.100769	1.0755	40.49	2.183
Eclipsed AA (hybrid)	5.17	–286.120689	1.0601	46.74	2.169
AB	4.31	–286.130213	1.0415	49.72	2.279

Supplementary Table 4 | The total DFTB energies, Lennard-Jones contributions (LJ), and the crystal stacking energies per monolayer as well as the corresponding HOMO-LUMO energy gap for HBC-COF

Stacking	*c* (Å)	Total DFTB Energy (a.u.)	LJ energy (a.u.)	Per unit crystal stacking energy (kcal mol^{-1})	HOMO–LUMO gap (eV)
Monolayer	–	–837.464224	3.8843	–	2.004
AA	3.54	–1676.652128	5.9878	135.20	1.553
Slipped AA (0.8 Å)	3.46	–1676.667025	6.0083	136.37	1.707
AB	3.29	–1676.108171	6.5496	92.54	1.795

Supplementary Table 5 | Porosity of HPB-COF treated with different solvents at different temperatures

Conditions	BET Surface Area (m^2 g^{-1}) for 25 °C	Pore Volume (cm^3 g^{-1}) for 25 °C	BET Surface Area (m^2 g^{-1}) for 100 °C	Pore Volume (cm^3 g^{-1}) for 100 °C
As synthesized	965	0.79	965	0.79
Hexane	867	0.70	858	0.75
THF	881	0.78	834	0.76
MeOH	860	0.73	805	0.69
Water	928	0.70	776	0.86
1 M NaOH	887	0.70	637	0.63

Supplementary Table 6 | Porosity of HBC-COF treated with different solvents at different temperatures

Conditions	BET Surface Area (m^2 g^{-1}) for 25 °C	Pore Volume (cm^3 g^{-1}) for 25 °C	BET Surface Area (m^2 g^{-1}) for 100 °C	Pore Volume (cm^3 g^{-1}) for 100 °C
As synthesized	469	0.29	469	0.29
Hexane	411	0.30	397	0.29
THF	460	0.28	436	0.31
MeOH	480	0.32	452	0.32
Water	431	0.29	394	0.29
1M NaOH	376	0.27	32	0.09
1 M HCl	21	0.08	21	0.06

Supplementary Methods

Materials. 1,3,5-Trimethylbenzene (mesitylene), anhydrous toluene, *o*-dichlorobenzene (*o*-DCB), anhydrous *n*-butanol (*n*-BuOH), chloroform ($CHCl_3$), dehydrated tetrahydrofuran (THF), hexane, dehydrated dichloromethane (CH_2Cl_2), dehydrated 1,4-dioxane (DOX), methanol (MeOH), ethanol (EtOH), pyridine, dimethyl sulfoxide-d_6 (99.9% D), anhydrous iron(III) chloride ($FeCl_3$), potassium carbonate (K_2CO_3), celite, sodium hydroxide (NaOH), hydrochloric acid (HCl), and acetic acid (AcOH) were purchased from Kanto Chemicals Co., Inc., Japan. Di-cobaltoctacarbonyl ($Co_2(CO)_8$), benzene-1,4-dicarbaldehyde (BDA), [bis(trifluoroacetoxy)iodo]benzene, 1,8-diazabicyclo [5.4.0] undec-7-ene (DBU), octanoyl chloride, and copper (I) iodide (CuI) were purchased from Sigma-Aldrich chemical company, Japan. Hexaphenylbenzene (HPB), anhydrous magnesium sulfate ($MgSO_4$), diethyl ether (Et_2O), Dimethylformamide (DMF), Iodine beads (I_2) and nitromethane ($MeNO_2$) were purchased from Wako Chemicals, Japan. Tetrakis(triphenylphosphine)palladium(0), bis(triphenylphosphine) palladium(II) dichloride, 4-(4,4,5,5-tetramethyl-1,3,2-dioxaborolan-2-yl)acetanilide, 4-iodoaniline, benzaldehyde, anhydrous sodium sulfate (Na_2SO_4), sodium chloride (NaCl) and trimethylsilylacetylene (TMSA) were purchased from Tokyo Kasai Co. (TCI), Japan. Chloroform-d_3 (D, 99.8%) was directly used from CIL, Inc. Japan.

$[NH_2]_6$HPB. $[NH_2]_6$HPB was prepared according to the reported methods as shown below.[1]

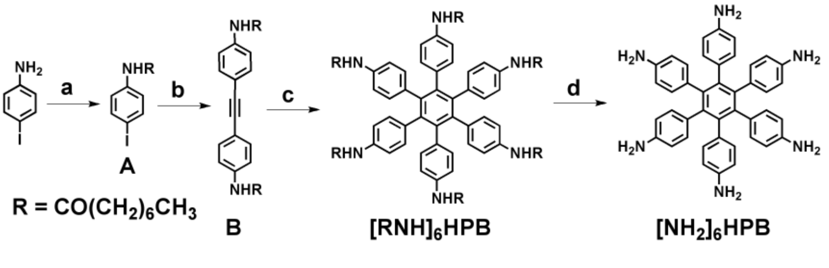

Reagents and conditions: (a) RCl (1.1 equiv. of 4-iodoaniline, R = $CO(CH_2)_6CH_3$), pyridine (1.1 equiv. of RCl), RT, 17 h; (b) $Pd(PPh_3)_2Cl_2$ (6 mol% of A), CuI (10 mol% of A), DBU (6 equiv. of A), trimethylsilylacetylene (0.5 equiv. of A), toluene/water (30/1), 60 °C, 72 h; (c) $Co_2(CO)_8$

(11 mol% of B), DOX (dry), Ar atmosphere, 110 °C, 72 h; (d) 6 M HCl, 90–100 °C, 5 days and 12 M HCl, 90 °C, additional 6 days.

Octanoylamino-4-iodobenzene (A). A mixture of 4-iodoaniline (10 g, 0.0456 mol), dehydrated dichloromethane (DCM, 160 mL), and pyridine (5 mL, pre-treated with anhydrous $MgSO_4$) in a 300-mL round bottle flask was stirred in ice-cooled water bath for 10 minutes and was then dropwise added with octanoyl chloride (9 mL, 0.053 mol) over 5 minutes. The mixture was stirred in the dark for 17 h at room temperature, reduced to 20 mL under vacuum, and added with hexane (200 mL), to yield large amount of crystalline white solid. The solid was filtered, washed with hexane (20 mL × 3), and dried under vacuum for 3 h. The solid was dissolve in 400 mL DCM and washed with water (200 mL × 2). The organic layers was collected and dried over anhydrous Na_2SO_4. The filtrate was evaporated and dried under vacuum for 6 h to yield 15 g octanoylamino-4-iodobenzene (95% yield). ^{1}H NMR ($CDCl_3$, δ (ppm), Supplementary Fig. 1): 7.58 (d, 2H, J = 7.0 Hz, ArH), 7.29 (d, 2H, J = 8.8 Hz, ArH), 7.2 (s, 1H, –CONH–), 2.32 (t, 2H, J = 7.3 Hz, J = 7.6 Hz, –CO–CH_2–), 1.59 (m, 2H, –CH_2–), 1.29 (m, 8H, –$(CH_2)_4$–), 0.86 (t, 3H, J = 6.7 Hz, J = 7.0 Hz, –CH_3). ^{13}C NMR ($CDCl_3$, δ (ppm)): 171.67, 137.96, 137.82, 121.72, 87.35, 37.90, 31.75, 29.30, 29.12, 25.62, 22.69, 14.17.

Bis[4-octanoylamino-phenyl]acetylene (B). A mixture of octanoylamino-4-iodobenzene (compound A, 10.2 g, 0.029 mol), toluene (300 mL), water (5 mL), and DBU (26 mL) in a 500-mL two-necked flask with condenser was bubbled with Ar for 45 minutes and added with $Pd(PPh_3)_2Cl_2$ (1.215 g, 6 mol% of compound A) and CuI (0.56 g, 10 mol% of compound A). The mixture was dropwise added with TMSA (2 mL, 0.0142 mol) via syringe over one minute under Ar flow. The system was degassed for 30 seconds at room temperature (twice), charged with Ar, and stirred at room temperature for 15 minutes and then at 60 °C for 72 h. After cooled down to room temperature, the collected solid was washed with DCM, $CHCl_3$, and water, respectively, dissolved in THF, and passed through celite. The THF solution was

evaporated to yield white pure product. The toluene solution part was passed through a celite plug, washed with water (200 mL × 2), 1 M HCl (100 mL × 2), brine (saturated NaCl solution, 200 mL × 2), and water (200 mL × 2), respectively. The toluene solution was dried over anhydrous sodium sulfate and was added with Et_2O (500 mL), to yield large amount of orange-brown precipitate. The solid was washed with Et_2O (25 mL × 3), DCM (until the eluent become colorless), $CHCl_3$ (50 mL × 2), and dried under vacuum for 6 h to yield compound B. The yield was 4.1 g (35% yield). ^{1}H NMR (d_6-DMSO, δ (ppm), Supplementary Fig. 2): 10.02 (s, 2H, –CONH–), 7.59 (d, 4H, J = 8.9 Hz, ArH), 7.40 (d, 4H, J = 6.7 Hz, ArH), 2.27 (t, 4H, J = 7.3 Hz, J = 7.6 Hz, –CO–CH_2–), 1.54 (t, 4H, J = 7.3 Hz, J = 7.0 Hz, –CH_2–), 1.24 (m, 16H, –$(CH_2)_4$–), 0.82 (t, 6H, J = 6.7 Hz, J = 7.0 Hz, –CH_3). ^{13}C NMR (d_6-DMSO, δ (ppm)): 172.07, 140.06, 132.38, 119.41, 117.12, 89.17, 36.99, 31.69, 29.14, 28.99, 25.58, 22.59, 14.48.

[RNH]$_6$HPB. A suspension of compound B (350 mg, 0.76 mmol) in dehydrated DOX (15 mL) in a 100-mL two-necked flask with condenser was bubbled with Ar for 45 minutes and was added with $Co_2(CO)_8$ (26 mg) in one portion. After 10-minute Ar bubbling, the mixture was degassed via three freeze-pump-thaw cycles, charged with Ar, and heated to reflux at 110 °C for 72 h. After cooled to room temperature, the mixture was added with Et_2O (80 mL), filtered, and washed with Et_2O (50 mL × 3). The residue was dissolved in minimum volume of DMF (20 mL), filtered through a celite plug (to remove the catalyst), and the solution was poured into water (300 mL). The precipitate was collected via filteration, washed with water, MeOH, and Et_2O, respectively, and dried under vacuum for 12 h to yield [RNH]$_6$HPB (280 mg). White powder in 80% yield. ^{1}H NMR (d_6-DMSO, δ (ppm), Supplementary Fig. 3): 9.49 (s, 6H, –CONH–), 7.05 (d, 12H, J = 7.6 Hz, ArH), 6.68 (d, 12H, J = 7.6 Hz, ArH), 2.12 (m, 12H, –CO–CH_2–), 1.18-1.44 (m, 60H, –$(CH_2)_5$–), 0.79 (m, 18H, –CH_3). ^{13}C NMR (d_6-DMSO, δ (ppm)): 171.46, 140.45, 136.89, 135.54, 131.57, 117.42, 36.93, 31.65, 29.23, 28.98, 25.45, 22.57, 14.43.

[NH$_2$]$_6$HPB. A mixture of [RNH]$_6$HPB (200 mg, 0.145 mmol) and 6M HCl (12 mL) in a 100-mL flask with condenser was stirred at 90-100 °C for 5 days under Ar. The mixture was added with conc. HCl (12 M, 6 mL) and heated to reflux for 6 days. After cooled down to room temperature, the solution was washed by Et_2O (50 mL × 2) and the aqueous layers were neutralized with NaOH solution (6 M) at 0 °C (ice-water bath). The resulting white precipitate was collected and thoroughly washed with water (50 mL × 3), MeOH (10 mL × 2), respectively. The solid was dried under vacuum for 12 h to yield [NH$_2$]$_6$HPB (90 mg). Light grey powder in 99% yield. ^{1}H NMR (d_6-DMSO, δ(ppm), Supplementary Fig. 4): 4.46 (s, 12H, –NH_2), 5.99 (d, 12H, J = 7.9 Hz, ArH), 6.31 (d, 12H, J = 7.9 Hz, ArH). ^{13}C NMR (d_6-DMSO, δ(ppm)): 145.02, 140.51, 131.98, 130.25, 113.13. MALDI-TOF-MS (Supplementary Fig. 9) for $C_{42}H_{36}N_6$ (calculated: 624.30), found: 623.50 ([M]$^+$).

[NH$_2$]$_6$HBC. [(NH$_2$)$_6$]HBC was synthesized using the route as shown below.[1,2]

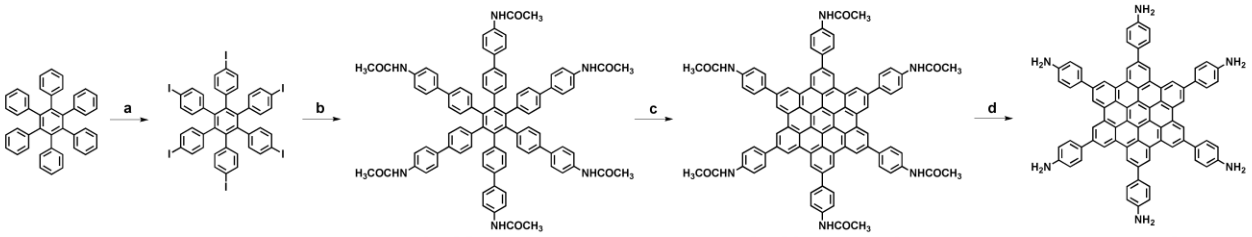

HPB HPBI$_6$ [CH$_3$CONH-HP]$_6$HPB [CH$_3$CONH-HP]$_6$HBC [NH$_2$]$_6$HBC

Reagents and conditions: (a) [bis(trifluoroacetoxy)iodo]benzene (3.37 equiv. of HPB), iodine (3.31 equiv. of HPB), CH_2Cl_2 (dry), Ar, room temperature, 24 h (in dark); (b) [4´-(4,4,5,5-tetramethyl-[1,3,2]dioxaborolan-2-yl)-phenyl]-acetanilide (8 equiv.), $(PPh_3)_4Pd(0)$ (10 mol%), K_2CO_3 (1.2 equiv.), dioxane/water (3/1 by vol.), Ar, 90-100 °C, 72 h; (c) $FeCl_3/MeNO_2$, CH_2Cl_2 (dry), Ar bubbled, RT, 24 h; (d) 6 M HCl, 90-100 °C, 72 h.

I$_6$HPB. A DCM (30 mL) solution of HPB (1g, 1.87 mmol) in a 200-mL flask was stirred under continuous Ar bubbling for 20 minutes and was added with [bis(trifluoroacetoxy)iodo]benzene (2.71 g, 6.30 mmol) in one portion. The solution was frozen with liquid nitrogen, added with iodine beads (1.57 g, 6.18 mmol), degassed via three freeze-pump-thaw cycles, and charged

with Ar. The mixture was stirred at room temperature in the dark for 24 h. The mixture was added with hexane (160 mL) to yield large amount of precipitate. The solid was filtered and washed with hexane (30 mL × 4). The white solid was dissolved in $CHCl_3$ (500 mL) and washed twice with $Na_2S_2O_4$ solution, brine, and water, respectively. The chloroform solution was passed through anhydrous Na_2SO_4 column and concentrated under vacuum until the white precipitate appeared. The residue was dissolved by heating at 100 °C and allowed to cool down at room temperature. The solution was kept undisturbed for two days for each temperature at 0 °C, – 4 °C and – 45 °C refrigerator, respectively, to yield large amount of colorless crystal. The crystal was collected by filtration, washed with cold chloroform (kept at – 4 °C for 6 h before use), and dried under vacuum. This crystallization process was repeated three times to yield 1.9 g of I_6HPB as a pure product. Colorless solid in 79% yield. ^{1}H NMR (d_6-DMSO, δ (ppm), Supplementary Fig. 5): 7.23 (d, 12H, J = 8.6 Hz, ArH), 6.45 (d, 12H, J = 8.6 Hz, ArH). ^{13}C NMR (d_6-DMSO, δ (ppm)): 139.56, 139.05, 136.40, 132.88, 92.04.

[CH_3CONH-HP]$_6$HPB. A mixture of I_6HPB (308.78 mg, 0.239 mmol), tetrakis(triphenylphosphine)palladium(0) (27.66 mg, 0.0239 mmol), K_2CO_3 (317.6 mg, 2.298 mmol), 4-(4,4,5,5-tetramethyl-1,3,2-dioxaborolan-2-yl)acetanilide (500 mg, 1.91 mmol), and dioxane/water (20 mL, 3/1 by vol.) in a 100-mL two-necked round bottle flask was purged with Ar for 30 minutes and stirred under reflux for three days. After cooled to room temperature, the mixture was added with a mixture of MeOH and water (50 mL, 1/1 by vol.). The precipitate was collected by filtration, washed with water, MeOH, THF and $CHCl_3$, and dried under vacuum to yield [CH_3CONH-HP]$_6$HPB as light grey solid (72% yield). ^{1}H NMR (d_6-DMSO, δ (ppm), Supplementary Fig. 6): 9.87 (s, 6H, –CONH–), 7.46 (d, 12H, J = 9.2 Hz, ArH), 7.36 (d, 12H, J = 8.8 Hz, ArH), 7.18 (d, 12H, J = 8.8 Hz, ArH), 6.98 (d, 12H, J = 8.0 Hz, ArH), 1.96 (s, 18H, –CH_3). ^{13}C NMR (d_6-DMSO-CD_3CN (5:1 v/v), δ (ppm)): 168.77, 140.50, 139.51, 139.12, 136.51,

134.04, 132.14, 126.78, 124.62, 119.64, 24.54. MALDI-TOF-MS for $C_{90}H_{72}N_6O_6$ (calculated: 1332.50), found: 1371.59 ($[M + K]^+$).

$[CH_3CONH\text{-}HP]_6HBC$. To a dehydrated dichloromethane suspension (50 mL) of $[CH_3CONH\text{-}HP]_6HPB$ (200 mg, 0.15 mmol) in a 100-mL two-necked round bottle flask under continuous Ar bubbling was slowly added with a nitromethane (5 mL) solution of anhydrous $FeCl_3$ (608.6 mg, 25 equiv.). The mixture was stirred at room temperature for 24 h and was quenched with excess MeOH. The precipitate was collected by filtration, washed with MeOH and CH_2Cl_2, and dried under vacuum. The solid was further purified using Soxhlet extraction in methanol for 72 h under Ar, cooled down at room temperature, and dried under vacuum to yield $[CH_3CONH\text{-}HP]_6HBC$ as orange powder (86% yield). MALDI-TOF-MS (Supplementary Fig. 9) for $C_{90}H_{60}N_6O_6$ (calculated: 1320.46), found: 1319.14 ($[M – H]^+$). FT-IR (υ/cm^{-1}): 3396.03 (sh), 3292.86, 3251.4, 3182.93 (sh) (–NH stretch), 3104.83, 3034.44, 1669.09 (–CONH–), 1597.73, 1518.67 (–NH bend), 1428.99, 1397.17, 1370.18, 1317.14, 1255.43 (C–N stretch), 1185.04, 1109.83, 1012.45, 964.23, 828.27, 723.17, 617.10, 561.18, 519.72.

$[NH_2]_6HBC$. A HCl (6 M, 30 mL) suspension of $[CH_3CONH\text{-}HP]_6HBC$ (150 mg, 0.114 mmol) in a 100-mL two-necked round bottle flask was refluxed for 72 h at 90-100 °C. After cooled to room temperature, the precipitate was collected by centrifugation, washed with water, and neutralized with an aqueous NaOH solution (1 M) at 0 °C. The precipitate was washed with water and MeOH (40 mL × 4), DMF (20 mL × 2), and MeOH/THF (1/1 by vol., 20 mL × 2) to yield $[NH_2]_6HBC$ as orange-red powder (96% yield). ^{1}H NMR (d_6-DMSO, δ (ppm), Supplementary Fig. 7): 5.09 (s, 12H, –NH_2), 6.94 (s, 12H, ArH), 7.55 (s, 12H, ArH), 8.41 (s, 12H, ArH). MALDI-TOF-MS (Supplementary Fig. 9) for $C_{78}H_{48}N_6$ (calculated: 1068.39), found: 1067.39 ($[M – H]^+$). FT-IR (υ/cm^{-1}): 3435.56 (sh), 3340.10, 3214.75 (sh) (–NH stretch), 1619.91 (–NH bend), 1605.45, 1517.70, 1370.17, 1277.60 (C–N stretch), 1182.15, 1102.12, 824.42.

HPB-Ph. An ethanol mixture (2 mL) of $[NH_2]_6$HPB (10 mg), benzaldehyde (0.1 mL), and AcOH (0.1 mL 6 M in a round bottle flask (25 mL) was stirred under reflux at 110 °C in Ar for two days. After cool down, the solvent was evaporated under vacuum at 60 °C to give solid, which was washed with EtOH (4 × 10 mL), and dried under vacuum at 80 °C for 12 h to yield HPB-Ph as light yellow powder (82% yield). ^{1}H NMR (d_6-DMSO, δ (ppm), Supplementary Fig. 8): 8.42 (s, 6H, –CH=N–), 7.79 (d, 12H, J = 6.0 Hz, ArH), 7.41 (m, 18H, ArH), 6.98 (d, 12H, J = 8.5 Hz, ArH), 6.85 (d, 12H, J = 8.2 Hz, ArH). MALDI-TOF-MS (Supplementary Fig. 9) for $C_{84}H_{60}N_6$ (calculated: 1152.49), found: 1152.66 (100%, $[M]^+$). FT-IR (υ/cm^{-1}): 1626.66 (–C=N– stretch), 1596.77, 1577.49, 1505.17, 1451.17, 1312.32, 1194.69, 1169.62, 1014.37, 884.20, 833.09, 761.74.

HBC-Ph. An ethanol mixture (2 mL) of $[NH_2]_6$HBC (10 mg), benzaldehyde (0.1 mL), and AcOH (0.1 mL 6 M) in a round bottle flask (25 mL) was stirred under reflux at 110 °C in Ar for two days. After cool down, the solvent was evaporated under vacuum at 60 °C to give solid, which was washed with EtOH (4 × 10 mL), and dried under vacuum at 80 °C for 12 h to yield HBC-Ph as orange powder (80% yield). MALDI-TOF-MS (Supplementary Fig. 9) for $C_{120}H_{72}N_6$ (calculated: 1596.58), found: 1595.46 (100%, $[M–H]^+$). FT-IR (υ/cm^{-1}): 1625.7 (–C=N– stretch), 1596.77, 1576.52, 1506.13, 1450.21, 1370.18, 1311.36, 1168.65, 1107.90, 829.24, 756.92.

NMR spectroscopy. ^{1}H and ^{13}C NMR spectra were measured on JEOL models JNM–ECS400 or JNM-ECA600 NMR spectrometer, where chemical shifts (δ in ppm) were determined with a residual proton of the solvent as standard.

Mass spectrometry. Matrix-assisted laser desorption ionization time-of-flight mass (MALDI-TOF MS) spectra were recorded on an Applied Biosystems BioSpectrometry model Voyager-DE-STR spectrometer in reflector or linear mode.

IR spectroscopy. Fourier transform infrared (FT–IR) spectra were recorded on a JASCO model FT–IR–6100 infrared spectrometer.

Microscopy. Field–emission scanning electron microscopy (FE–SEM) was performed on a JEOL model JSM–6700 operating at an accelerating voltage of 5.0 kV. The sample was prepared by drop-casting THF suspension onto mica substrate and then coated with gold. High-resolution transmission electron microscopy (HR–TEM) images were obtained on a JEOL model JEM–3200 microscopy at an accelerating voltage of 300 kV with an electron beam of λ = 0.00197 nm. The samples were prepared by drop-casting sonicated THF suspensions of COFs onto a copper grid and the time duration for the image snap shot is 0.7 sec.

Electronic absorption spectroscopy. Electronic absorption spectra were recorded on a JASCO model V-670 spectrometer equipped with integration sphere model IJN–727.

X-ray diffraction. X-ray diffraction (XRD) data were recorded on a Rigaku model RINT Ultima III diffractometer by depositing powder on glass substrate, from 2θ = 1.5° up to 60° with 0.02° increment. Before X–ray measurement all COFs were dried under vacuum for 12 h.

Thermogravimetric analysis. Thermogravimetric analysis (TGA) was performed on a METTLER TOLEDO instrument (model TGA/SDTA851^{e}) under nitrogen atmosphere.

Nitrogen sorption isotherm measurements. Nitrogen sorption was measured at 77 K with a Bel Japan Inc. model BELSORP–mini II analyzer. Before measurement, the samples were degassed in vacuum at 120 °C for 6 h and samples of about 30 mg were used for each measurement. The Brunauer–Emmett–Teller (BET) method was utilized to calculate the specific surface areas

(ranged from P/P_0 0.005-0.1 with coefficient of determination R^2 = 0.9998-1.0000). By using the non–local density functional theory model (NLDFT), the pore size and volume were derived from the sorption curve.

Stability test. To examine the chemical stability, we dispersed the COF samples in different solvents such as hexane, THF, MeOH, water, aqueous HCl (1M) and NaOH (1 M) solutions and kept at 25 °C or boiling temperatures (heating at 100 °C) for 24 h, respectively. To test HPB-COF in water and aqueous NaOH solution, we moistened the powder samples with minimum volume of MeOH (*e.g.* 0.2 mL for 30 mg sample) for a better dispersed in the aqueous media. The samples for treatment at 100 °C were degassed via three freeze-pump-thaw cycles and sealed to keep at 100 °C for 24 h, while the experiments for treatment at 25 °C were performed under air. After treatment in organic solvents, the samples were collected via filtration, washed with THF and dispersed in THF for 6 h, centrifuged, collected, dried under vacuum for 12 h, and subjected to XRD and nitrogen sorption measurements. After treatment with HCl, the samples were washed with water, NaOH (1 M), water, MeOH and THF, respectively and then dispersed in THF for 6 h, centrifuged, collected, dried under vacuum for 12 h, and subjected to XRD and nitrogen sorption measurements. For the NaOH (1 M) treated samples, the remaining solid was washed with water, MeOH and THF, respectively and then dispersed in THF for 6 h, centrifuged, collected, dried under vacuum for 12 h, and subjected to XRD and nitrogen sorption measurements.

Theoretical surface area calculations. Adsorption isotherm simulations were performed under the 'Sorption' module of Materials Studio[3]. Metropolis Monte Carlo[4] method was utilized for calculation of the nitrogen adsorption in the framework under 40 fugacity steps in a logarithmic scale (10^{-5} to 100 kPa), the COMPASS force field was selected for the energy calculation. All simulations included random insertion/deletion, translation, and rotation moves of molecules with equal probabilities. Atoms in COFs were fixed at their crystallographic positions. An LJ cutoff distance of 13 Å was used. The Ewald & Groups technique was used to compute the

electrostatic interactions. All GCMC simulations included a 1,000,000-cycle equilibration period followed by a 10,000,000-cycle production run. The 2 × 2 × 2 unit cells of HPB-COF and 1 × 1 × 2 unit cells of HBC-COF were used for simulations of the N_2 adsorption isotherms at 77 K[5,6]. BET surface areas were determined from the calculated isotherms by the same method used for treating experimental data at the low relative pressure range of 0.005 to 0.05[7].

Pawley refinements. Molecular modeling and Pawley refinement were carried out using Reflex, a software package for crystal determination from XRD pattern, implemented in MS modeling version 4.4 (Accelrys Inc.)[3]. Initially, unit cell dimensions for both COFs were taken from the DFTB calculation (see the text below) and the space group for triangular crystal system was selected as P_1. We performed Pawley refinement to optimize the lattice parameters iteratively until the R_{WP} value converges. The pseudo–Voigt profile function was used for whole profile fitting and Berrar–Baldinozzi function was used for asymmetry correction during the refinement processes. The final R_{WP} and R_P values were 8.67% and 6.53% for HPB–COF and 9.04% and 7.13% for HBC–COF, respectively. The corresponding unit cell parameters are $a = b = 21.6$ Å, $c = 4.7$ Å, $\alpha = \beta = 90°$, and $\gamma = 120°$, for HPB–COF and $a = b = 30.2$ Å, $c = 3.4$ Å, $\alpha = \beta = 90°$, and $\gamma = 120°$, for HBC–COF.

DFTB calculations. Optimized molecular structure of the monolayer and stacked isomers were determined using the density-functional tight-binding (DFTB) method including Lennard-Jones (LJ) dispersion. The corresponding LJ and crystal stacking energies as well as the HOMO-LUMO energy gaps were computed. The calculations were carried out with the DFTB+ program package version 1.2[8]. DFTB is an approximate density functional theory method based on the tight binding approach and utilizes an optimized minimal LCAO Slater-type all-valence basis set in combination with a two–center approximation for Hamiltonian matrix elements. The Coulombic interaction between partial atomic charges was determined using the self-consistent charge (SCC) formalism. LJ type dispersion was employed in all calculations to describe van der Waals (vdW) and π-stacking interactions. The lattice dimensions were optimized simultaneously

with the geometry. Standard DFTB parameters for X-Y element pair (X, Y = C, H, and N) interactions were employed from the mio-0-1 set[9]. The single layer model system consisted of 114 atoms for HPB-COF and 648 atoms for HBC-COF, where a 2 × 2 supercell was employed. The cell had an optimal lattice constant of $a = b = 21.57$ Å for HPB-COF and $a = b = 60.28$ Å for HBC-COF with an angle of 60° between the vectors. Using the optimized monomer, different stacking configurations, such as eclipsed AA (hybrid), slipped AA by 0.8 Å in the *a* and *b* directions, and staggered AB were optimized. The third dimension of the lattice, *c* was initialized for all structures at 5.0 Å for HPB-COF and at 3.5 Å for HBC-COF.

Supplementary References

1. Kobayashi, K. *et al*. Syntheses of hexakis(4-functionalized–phenyl)benzenes and hexakis[4-(4-functionalized-phenylethynyl)phenyl]benzenes directed to host molecules for guest-inclusion networks. *J. Org. Chem.* **70**, 749-752 (2005).
2. Wu, J., Watson, M. D., Zhang, L., Wang, Z. & Müllen, K. Hexakis(4-iodophenyl)-peri-hexabenzocoronene – a versatile building block for highly ordered discotic liquid crystalline materials. *J. Am. Chem. Soc.* **126**, 177-186 (2004).
3. Accelrys, Material studio release notes, Release 4.4, Accelrys Software, San Diego (2008).
4. Metropolis, N., Rosenbluth, A. W., Rosenbluth, M. N., Teller, A. H. & Teller, E. Equation of State Calculations by Fast Computing Machines. *J. Phys. Chem.* **21**, 1087-1092, (1953).
5. Feng, X. *et al.* An Ambipolar Conducting Covalent Organic Framework with Self-Sorted and Periodic Electron Donor-Acceptor Ordering. *Adv. Mater.* **24**, 3026-3031, (2012).
6. Rankin, R. B., Liu, J., Kulkarni, A. D. & Johnson, J. K. Adsorption and Diffusion of Light Gases in ZIF-68 and ZIF-70: A Simulation Study. *J. Phys. Chem.* C **113**, 16906-16914, (2009).
7. Walton, K. S. & Snurr, R. Q. Applicability of the BET method for determining surface areas of microporous metal-organic frameworks. *J. Am. Chem. Soc.* **129**, 8552-8556, (2007).
8. Aradi, B., Hourahine, B. & Frauenheim, T. DFTB+, a sparse matrix–based implementation of the DFTB method. *J. Phys. Chem.* A **111**, 5678-5684 (2007).
9. http://www.dftb.org
